# Bromodomain-containing protein 9 promotes the growth and metastasis of human hepatocellular carcinoma by activating the TUFT1/AKT pathway

**DOI:** 10.1038/s41419-020-02943-7

**Published:** 2020-09-09

**Authors:** Changwei Dou, Liankang Sun, Liang Wang, Jian Cheng, Weiding Wu, Chengwu Zhang, Qiuran Xu, Kangsheng Tu, Jie Liu

**Affiliations:** 1 Department of Hepatopancreatobiliary Surgery & Minimally Invasive Surgery, Zhejiang Provincial People’s Hospital (People’s Hospital of Hangzhou Medical College), 310014 Hangzhou, China; 2 Department of Hepatobiliary Surgery, The First Affiliated Hospital of Xi’an Jiaotong University, 710061 Xi’an, China; 3 Key Laboratory of Tumor Molecular Diagnosis and Individualized Medicine of Zhejiang Province, Zhejiang Provincial People’s Hospital (People’s Hospital of Hangzhou Medical College), 310014 Hangzhou, China

**Keywords:** Liver cancer, Oncogenes

## Abstract

Bromodomain-containing protein 9 (BRD9) has a critical role in human squamous cell lung cancer, acute myeloid leukemia, and malignant rhabdoid tumors. However, the expression and biological role of BRD9 in hepatocellular carcinoma (HCC) is poorly understood. In this study, BRD9 expression was found to be elevated in HCC through data mining of public databases. Next, we confirmed that the expression of BRD9 was increased in HCC tissues compared with that in adjacent non-tumor tissues. The upregulated level of BRD9 was also observed in HCC cells in comparison to LO2 cells. The increased BRD9 expression was correlated with unfavorable clinicopathological features. A high level of BRD9 predicted a poorer overall survival and disease-free survival of HCC patients. Functionally, BRD9 overexpression facilitated the proliferation, migration, invasion, and epithelial–mesenchymal transition (EMT) of Hep3B cells. Conversely, either BRD9 depletion or pharmacological inhibition of BRD9 resulted in the reduced proliferation and invasiveness of HCCLM3 cells. In addition, the BRD9 knockdown restrained the growth and metastasis of HCCLM3 cells in vivo. Mechanistically, BRD9 positively regulated TUFT1 expression and AKT activation in HCC cells. ChIP-qPCR analysis indicated that BRD9 promoted the binding of P300 acetyltransferase to the TUFT1 promoter and epigenetically regulated TUFT1 expression by increasing H3K27Ac in the promoter. Notably, either TUFT1 knockdown or AKT inhibitor (MK2206) abrogated the promoting effects of BRD9 on the proliferation, migration, invasion, and EMT of Hep3B cells. The forced expression of TUFT1 abolished the effects of BRD9 knockdown on the growth and metastasis of HCCLM3 cells. Altogether, these data indicate that BRD9 promotes the growth and metastasis of HCC cells by activating the TUFT1/AKT pathway and may serve as a promising biomarker and therapeutic target for HCC.

## Introduction

Hepatocellular carcinoma (HCC) is ranked as the sixth most common malignancy and the third leading cause of cancer-related mortality, with ~780,000 newly diagnosed cases and 750,000 deaths annually^1,2^. The pathogenesis of HCC is a complex process that includes sustained inflammatory damage and fibrotic deposition. The risk of HCC is markedly increased in patients with cirrhosis and has been recognized as a leading cause of death among these patients^3^. Despite remarkable progress in imaging diagnosis, surgical techniques, chemotherapy, and targeted therapy in recent years, the 5-year survival rate of HCC is still lower than 20% due to rapid tumor growth and a high incidence of tumor recurrence and metastasis^4^. Therefore, a better understanding of the molecular mechanisms underlying the progression of HCC can facilitate the development of novel targeted drugs and result in the improvement of overall prognosis.

Recently, bromodomain-containing protein 9 (BRD9) was identified to be a component of the SWI-SNF chromatin-remodeling complex, which participates in gene transcription, DNA repair, and cell differentiation^5^. BRD9, which harbors a single bromodomain (BD), acts as an epigenetic reader that recognizes acetylated lysines in histones and nonhistone proteins^6,7^. In AML cells, BRD9 binds to BRG1 and is essential for supporting enhancer-mediated MYC expression^8,9^. Inhibitors of BRD9 suppressed the proliferation of mouse and human AML cells^8^. BRD9 was also found to promote AML cell survival primarily by regulating the SOCS3-STAT5 pathway^10^. In MCF-10A cells, BRD9 was an important mediator of mutant PIK3CA/KRAS-driven oncogenic transformation^11^. In basal cell carcinoma (BCC), BRD9 was upregulated in both murine and human HHi (Hedgehog inhibitors)-resistant BCC. An inhibitor of BRD9 reversed HHi resistance and restored the vismodegib sensitivity of BCC^12^. BRD9 inhibition in rhabdoid tumors resulted in decreased cell proliferation, G1-arrest, and apoptosis^13^. However, the role of BRD9 in HCC remains unknown.

In this study, we found that BRD9 expression was elevated in HCC tissues and cell lines. BRD9 promoted the proliferation, migration, invasion, and epithelial–mesenchymal transition (EMT) of HCC cells. The knockdown of BRD9 suppressed the growth and metastasis of HCC in vivo. Mechanically, we found that BRD9 could promote the binding of P300 to the TUFT1 promoter and epigenetically turned on the expression of TUFT1 by increasing H3K27Ac in the TUFT1 promoter. Activation of the TUFT1/AKT pathway was critical for the oncogenic functions of BRD9 in HCC. These findings demonstrate the role of BRD9 in HCC and may provide a novel biomarker and therapeutic target for HCC.

## Materials and methods

### Patients’ cohort, clinical specimens, and data mining of online databases

A total of 110 HCC patients who underwent curative resection in the Department of Hepatobiliary Surgery, the First Affiliated Hospital of Xi’an Jiaotong University (Xi’an, China), were enrolled in this study. None of the patients received any chemotherapy or radiotherapy before the operations. The median follow-up of the patients was 34.3 months (1–86 months). The diagnosis of all HCC cases was validated by pathological examinations. Clinical specimens, including HCC tissues and adjacent non-tumor tissues, were collected. These samples were stored at −80 °C for RNA and protein extraction or formalin-fixed and embedded in paraffin. Written informed consent was obtained from each patient. The study protocol was approved by the research ethics committee of the First Affiliated Hospital of Xi’an Jiaotong University. The clinical characteristics of the HCC cohort are presented in Supplementary Table 1. The expression status of BRD9 in different types of human cancers and the corresponding normal tissues was obtained from the Gene Expression Profiling Interactive Analysis (GEPIA) website^14^ (http://gepia.cancer-pku.cn/index.html). The expression data of BRD9 in the TCGA database and GEO databases (GSE 14323, GSE 14520, GSE1898, GSE32649, and GSE 6764) were obtained from the Oncomine website^15^ (https://www.oncomine.org/). The statistical threshold used for determining the overexpression of BRD9 in the tumor samples vs the normal tissue in these public datasets was *P* < 0.05.

### Antibodies

The antibodies used in this study included: anti-BRD9 (Active Motif, Carlsbad, CA, USA, #61537), anti-TUFT1 (Abcam, Cambridge, MA, USA, ab184949), anti-E-cadherin (Cell Signaling Technology, Beverly, MA, USA, #3195), anti-N-cadherin (Cell Signaling Technology, #14215), anti-vimentin (Cell Signaling Technology, #5741), anti-H3K27Ac (Abcam, ab4729), anti-H3K14Ac (Abcam, ab52946), anti-H3K9Ac (Abcam, ab4441), anti-P300 (Abcam, ab10485), anti-AKT (Cell Signaling Technology, #4691), anti-phospho-AKT (Ser-473, Cell Signaling Technology, #4060), anti-MYC (Cell Signaling Technology, #9402), and anti-GAPDH (Santa Cruz Biotechnology, Dallas, TX, USA, sc-47724).

### Cell culture

The immortalized hepatocyte cell line LO2 and five HCC cells lines (HepG2, Hep3B, Huh7, MHCC97H, and HCCLM3) were obtained from the Cell Bank, Type Culture Collection, Chinese Academy of Sciences (Shanghai, China) and passed the test of DNA profiling (STR) in the year 2016. The cells were maintained in Dulbecco’s modified Eagle’s medium (DMEM; Gibco, USA) supplemented with 10% fetal bovine serum (FBS; Gibco, USA), 100 units/mL penicillin and 100 μg/mL streptomycin (Sigma, St-Louis, MO, USA). All cell cultures were kept in a humidified cell incubator with 5% CO_2_ at 37 °C. To inhibit the activation of the AKT pathway, the cells were starved overnight and treated with MK2206 (10 µM)^16^. For BRD9 inhibition, the cells were starved overnight and treated with I-BRD9 (5 µM)^13^. All cells were routinely tested for mycoplasma contamination with a MycoAlert Detection Kit (Lonza).

### Viral transduction and cell transfection

Brd9 shRNA (Addgene #75138) underwent enzymatic digestion with MluI and XhoI and was then inserted into the pGipZ lentiviral vector (NEB Quick Ligation Kit). Nontargeting hairpin shRNA in pGipZ was used as a control. The retroviral vector pMMP-BRD9 was generated by inserting the BRD9 cDNA into the pMMP vector. For retroviral packaging, the pMMP-BRD9 plasmid was transfected into 293T cells using Effectene (Qiagen, Valencia, CA, USA) along with the pMD.MLV plasmid and the pVSV.G plasmid. Viral supernatants were collected 72 h after transfection. Viral transduction was performed by incubating cells with the viral supernatant supplemented with polybrene (8 μg/mL) overnight at 37 °C. Further experiments were performed 48 h after viral transduction. The efficacy of viral transduction was confirmed by qRT-PCR and western blot. TUFT1 shRNA, P300 shRNA, TUFT1 vector, the corresponding NC shRNA, or the corresponding control vector was transfected into HCC cells as previously described^16,17^. HCC cells were subjected to western blotting and qRT-PCR to confirm the efficacy of TUFT1 or P300 knockdown.

### Quantitative real-time PCR (qRT-PCR)

RNA extraction and qRT-PCR were performed as previously described^18^. 18S RNA was used as an internal control. Primer sequences for BRD9, TUFT1, E-cadherin, N-cadherin, vimentin, and 18S RNA are listed in Supplementary Table 2.

### Immunohistochemistry (IHC) staining

Immunohistochemistry (IHC) staining was performed as described previously^18^. The following antibodies were used in the IHC staining: BRD9 antibody (1:50) and TUFT1 antibody (1:50). The intensity of IHC staining was graded as per the following criteria: 0, negative staining; 1, weak staining; 2, medium staining; and 3, strong staining. The percentage of positive staining was graded as per the following criteria: 0, <5%; 1, 5–25%; 2, 25–50%; 3, 50–75%; and 4, 75–100%. The IHC score of each slide was calculated by multiplying the scores of the staining intensity and the percentage of positive staining.

### Western blotting

Protein extraction and western blotting were performed as described previously^16^. The following primary antibodies were used in this study: anti-BRD9 (1:1000), anti-TUFT1 (1:1000), anti-P300 (1:500), anti-E-cadherin (1:1000), anti-N-cadherin (1:500), anti-vimentin (1:1000), anti-p-AKT (1:1000), anti-AKT (1:1500), and anti-GAPDH (1:1500). A densitometric measurement was performed by using ImageJ software and normalized to GAPDH^19^.

### Proliferation assay

The cell viability of HCC cells was evaluated by the MTT assay. The 3-(4,5-dimethylthiazol-2-yl)-2,5-diphenyl tetrazolium bromide (MTT, Roche, USA) was used for the MTT assay as described previously^16^. EdU and colony formation assays were performed for the evaluation of cell proliferation. For the EdU assay, a cell proliferation ELISA, the Cell-Light™ EdU Apollo®488 In Vitro Imaging Kit (#C10310-3, RiboBio Co., LTD, Guangzhou, China), was used as previously described^20^. For the colony formation assay, HCC cells (1 × 10^3^) were seeded in 6-well plates and cultured for 2 weeks in DMEM medium with 10% FBS. The cell colonies containing more than 30 cells were counted as single colonies^20^.

### Transwell assay

The migration and invasion ability of the HCC cells was evaluated by a Transwell assay. In general, serum-starved HCC cells were resuspended in DMEM and seeded into the upper chamber. For the invasion assay, 150 µL Matrigel (1:8 dilution in serum-free DMEM) was added onto the membrane of the upper chamber before cell seeding. DMEM containing 20% FBS (600 µL) was added into the bottom chamber. Twenty-four hours after cell seeding, the HCC cells that migrated or invaded through the membrane of the upper chamber were stained with crystal violet. The cell number of the migrated or invaded cells was counted.

### Chromatin immunoprecipitation (ChIP)

ChIP was performed to investigate whether BRD9 affected the histone acetylation status in the promoter of the TUFT1 gene and the engagement of P300 to the TUFT1 promoter. The HCC cells were fixed with 1% formaldehyde. ChIP assays were performed following the protocol of the EZ-Magna-ChIP HiSens kit (Millipore, 17-10461). Nuclear extracts were subjected to sonication on wet ice. The samples were sheared during three rounds of ten cycles of 30 s ON/30 s OFF with the Bioruptor® PLUS at the HIGH power setting (position H). Magnetic protein A/G beads were used for the immunoprecipitation (IP) of cross-linked protein/DNA. Antibodies against H3K9Ac (1:25), H3K14Ac (1:25), H3K27Ac (1:25), or P300 (1:50) or control IgG (1:25) were used to immunoprecipitate the sheared DNA. The precipitated DNAs containing fragments of the TUFT1 promoter were detected by qRT-PCR using seven pairs of walking primers along the TUFT1 promoter region^16^. The sequences of the seven pairs of walking primers are listed in Supplementary Table 2. The fold enrichment was relative to the input DNA.

### In vivo growth and metastasis experiments

BALB/c nude mice (male, 4–6 weeks old) were randomly grouped for animal experiments (*n* = 6 mice per group). HCCLM3 cells with a BRD9 knockdown (HCCLM3-BRD9 shRNA) or negative control HCCLM3 cells (HCCLM3-NC shRNA) were employed for the tumor growth and metastasis experiments in nude mice. The subcutaneous injection experiment and tail-vein injection experiment were performed to evaluate the growth and metastasis of HCC cells as previously described^16^. Subcutaneous tumor tissues were dissected out for IHC staining of Ki67 (1:100), E-cadherin (1:50), and vimentin (1:100). The lungs of the nude mice were embedded in paraffin for H&E staining to identify the metastatic nodules. Animal protocols were approved by the Institutional Animal Care and Use Committee of Xi’an Jiaotong University.

### Statistical analysis

The data in this study are shown as the mean ± S.E.M. At least three independent replicates were used to calculate S.E.M. Statistical analysis was performed by GraphPad Prism 5 software (San Diego, CA, USA) and the SPSS statistical package (Chicago, IL, USA). A two-tailed Student’s *t*-test, Pearson *χ*^2^ test, Kaplan–Meier plot, log-rank test, Spearman’s rank correlation coefficient, or ANOVA was used to determine the significant difference between groups. A *P* < 0.05 was statistically significant.

## Results

### BRD9 expression is increased in HCC

We determined the expression of BRD9 using the GEPIA database (http://gepia.cancer-pku.cn/). Among 23 types of human cancers, HCC tissues showed an obvious elevation in BRD9 expression, compared with the corresponding normal tissues (Supplementary Fig. 1A). Next, we investigated the expression of BRD9 in HCC based on the TCGA database and Gene Expression Omnibus (GEO) datasets. As shown in Supplementary Fig. 1B, the data from the TCGA database and 5 GEO datasets consistently demonstrated that the expression of BRD9 in HCC tissues was significantly higher than that in non-tumor liver tissues (*P* < 0.05, Supplementary Table 3). The IHC data from the Human Protein Atlas (https://www.proteinatlas.org/) revealed that the intensity of BRD9 staining in HCC tissues was stronger than that in normal liver tissues (Supplementary Fig. 2).

**Fig. 1:**
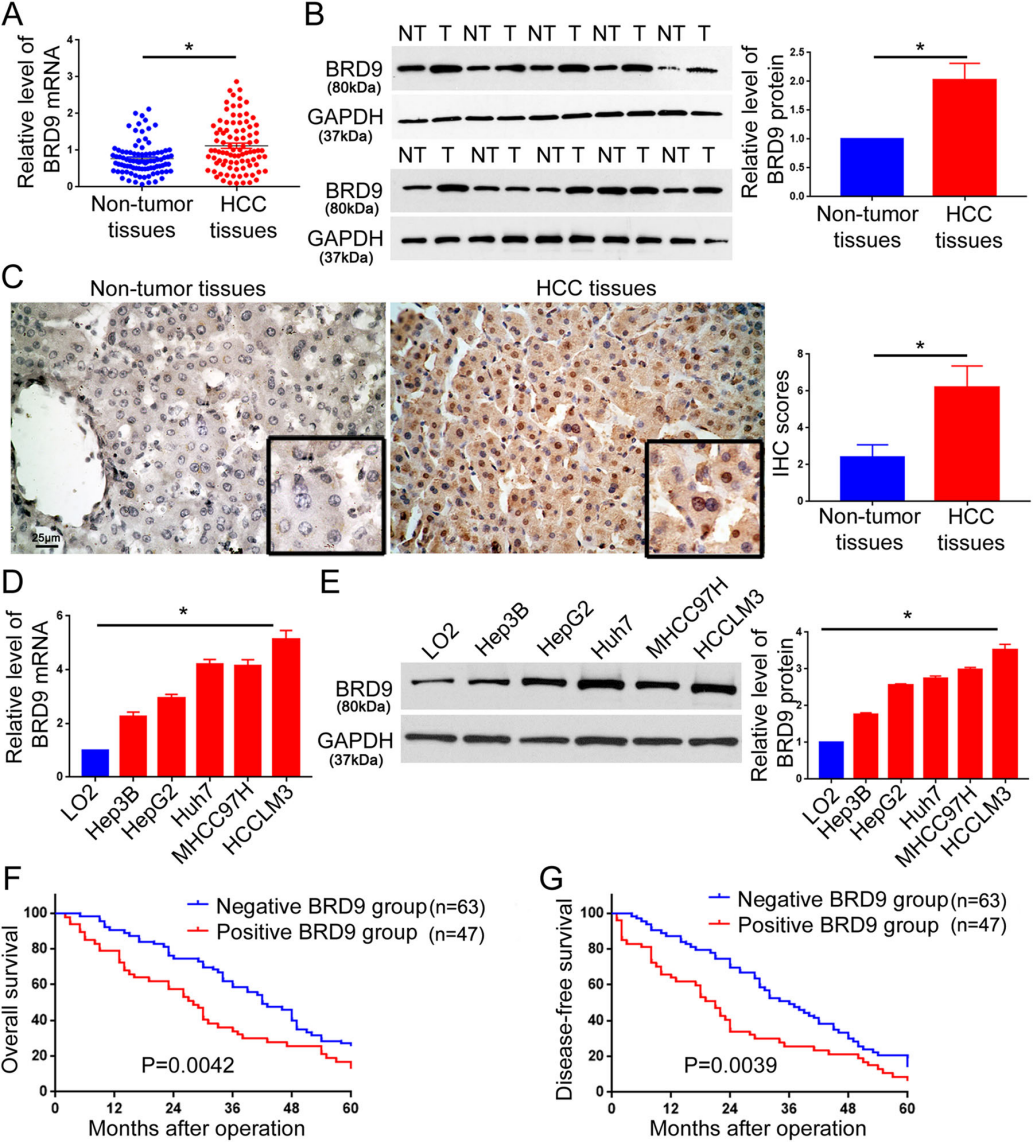
The expression and prognostic significance of BRD9 in HCC. **a** qRT-PCR for BRD9 mRNA was performed in 110 pairs of HCC and matched adjacent non-tumor liver tissues. *P < 0.05 by *t*-test. **b** Western blot analysis of BRD9 was performed in 20 pairs of HCC tissues and matched adjacent non-tumor liver tissues. *P < 0.05 by *t*-test. **c** Representative IHC staining of BRD9 in HCC tissues and adjacent non-tumor liver tissues. IHC scores for BRD9 between HCC and adjacent non-tumor liver tissues were compared. Scale bar: 25 μm. *n* = 110, *P < 0.05 by *t*-test. **d** qRT-PCR and **e** western blotting were performed for BRD9 expression in the immortalized hepatocyte cell line LO2 and five HCC cell lines (HepG2, Hep3B, Huh7, MHCC97H, and HCCLM3). *n* = three independent experiments, *P < 0.05 by ANOVA. **f** Overall survival and **g** disease-free survival were compared between HCC cases with positive BRD9 staining and those with negative BRD9 staining.

To further validate the data from online databases, we quantified the expression of BRD9 in 110 pairs of HCC specimens and adjacent non-tumor liver tissues. The qRT-PCR assay showed that compared with adjacent non-tumor tissues, the level of BRD9 mRNA in HCC tissues was significantly elevated (Fig. 1a, *P* < 0.05). Western blot and IHC assays confirmed that the level of BRD9 protein was increased in HCC tissues (Fig. 1b, c, *P* < 0.05). Furthermore, we measured the levels of BRD9 in HCC cell lines (Hep3B, Huh7, HepG2, MHCC97H, and HCCLM3). Compared with the immortalized hepatocyte cell line LO2, the HCC cell lines showed elevated levels of BRD9 mRNA and protein (Fig. 1d, e, *P* < 0.05). Altogether, these data verify that BRD9 expression is significantly upregulated in HCC.

### An elevated BRD9 level confers poor clinical features and prognosis in HCC patients

Next, we explored whether the expression of BRD9 was associated with the clinical features and prognosis in HCC patients. As shown in Supplementary Table 1, positive staining of the BRD9 protein in HCC tissues was associated with a tumor size ≥ 5 cm (*P* = 0.027), the occurrence of vascular infiltration (*P* = 0.003), and an advanced TNM stage (*P* = 0.003). More importantly, HCC patients with positive staining for BRD9 had a significantly decreased overall survival (Fig. 1f, *P* = 0.0042) and disease-free survival (Fig. 1g, *P* = 0.0039). Consistently, these data in the TCGA database demonstrate that an elevated level of BRD9 in HCC tissues is correlated with a worse overall survival (Supplementary Fig. 3A, *P* = 0.00061) and disease-free survival (Supplementary Fig. 3B, *P* = 0.038).

### BRD9 promotes the proliferation, migration, invasion, and EMT of HCC cells in vitro

**Fig. 2:**
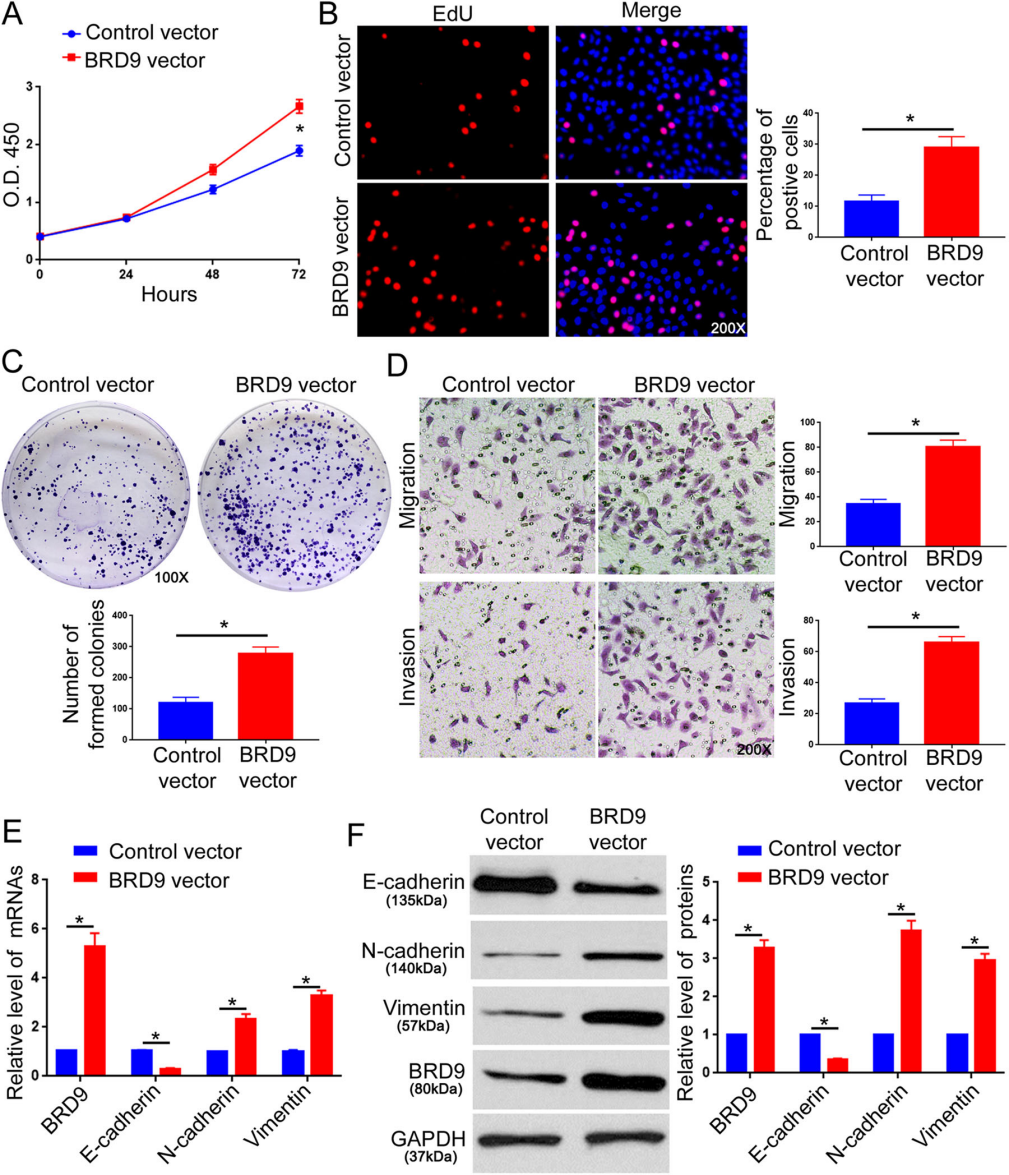
BRD9 overexpression promotes the proliferation, migration, invasion, and EMT of Hep3B cells. **a** The MTT assay was performed to evaluate the effect of BRD9 overexpression on the viability of Hep3B cells. *n* = three independent experiments, *P < 0.05 by ANOVA. **b** The EdU assay was performed to evaluate the effect of BRD9 overexpression on the proliferation of Hep3B cells. *n* = five randomly selected fields of three independent experiments, *P < 0.05 by *t*-test. **c** The colony formation assay was performed to evaluate the effect of BRD9 overexpression on the proliferation of Hep3B cells. *n* = three independent experiments, *P < 0.05 by *t*-test. **d** The Transwell assay was used to investigate the effect of BRD9 overexpression on the migration and invasion of Hep3B cells. *n* = five randomly selected fields of three independent experiments, *P < 0.05 by *t*-test. **e** qRT-PCR and **f** western blotting were performed to evaluate the effect of BRD9 overexpression on the expression of EMT markers (E-cadherin, N-cadherin, and vimentin). *n* = three independent experiments, *P < 0.05 by *t*-test.

**Fig. 3:**
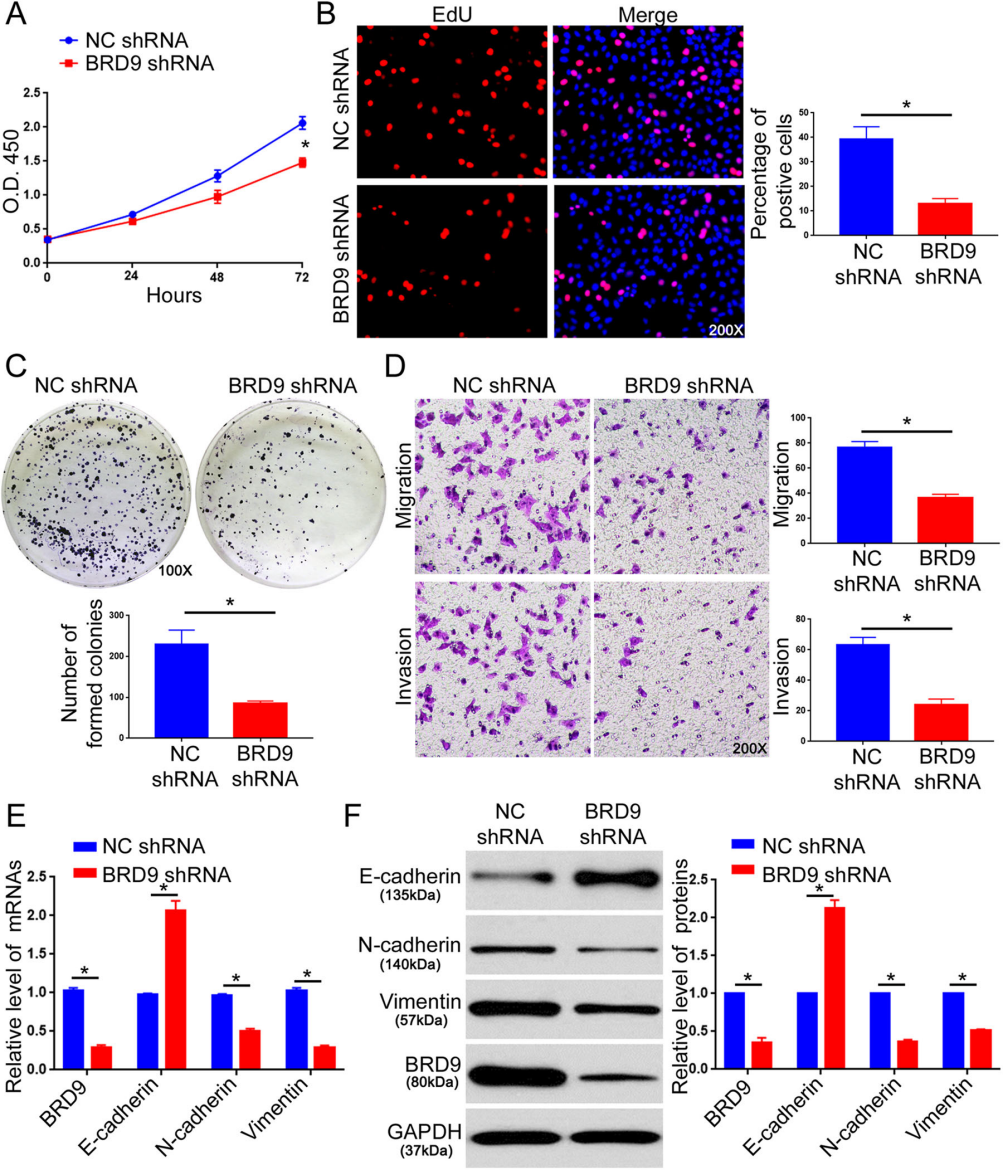
BRD9 knockdown inhibits the proliferation, migration, invasion, and EMT of HCCLM3 cells. **a** The MTT assay was performed to evaluate the effect of BRD9 knockdown on the viability of HCCLM3 cells. *n* = three independent experiments, *P < 0.05 by ANOVA. **b** The EdU assay was performed to evaluate the effect of BRD9 knockdown on the proliferation of HCCLM3 cells. *n* = five randomly selected fields of three independent experiments, *P < 0.05 by *t*-test. **c** The colony formation assay was performed to evaluate the effect of BRD9 knockdown on the proliferation of HCCLM3 cells. *n* = three independent experiments, *P < 0.05 by *t*-test. **d** The Transwell assay was used to investigate the effect of BRD9 knockdown on the migration and invasion of HCCLM3 cells. *n* = five randomly selected fields of three independent experiments, *P < 0.05 by *t*-test. **e** qRT-PCR and **f** western blotting were performed to evaluate the effect of BRD9 knockdown on the expression of EMT markers (E-cadherin, N-cadherin, and vimentin). *n* = three independent experiments, *P < 0.05 by *t*-test.

Since BRD9 showed a relatively lower expression in Hep3B cells and a higher level in HCCLM3 cells among the five HCC cell lines (Fig. 1d, e), Hep3B cells with a stable BRD9 overexpression (Hep3B-BRD9, Supplementary Fig. 4A, B) and HCCLM3 cells with a stable BRD9 knockdown (HCCLM3-BRD9 shRNA, Supplementary Fig. 4C, D) were established. MTT and EdU assays showed that overexpression of BRD9 increased the viability and proliferation of Hep3B cells (Fig. 2a, b, *P* < 0.05). The colony formation assay revealed that the forced expression of BRD9 increased the colony formation potential of Hep3B cells (Fig. 2c, *P* < 0.05). The Transwell assays demonstrated that BRD9 overexpression enhanced the migration and invasion of Hep3B cells (Fig. 2d, *P* < 0.05). Since EMT was critical for the metastasis of cancer cells^21,22^, we explored whether BRD9 affected the EMT of HCC cells. As expected, BRD9 overexpression led to the decreased expression of E-cadherin and elevated levels of N-cadherin and vimentin (Fig. 2e, f, *P* < 0.05). Conversely, BRD9 knockdown repressed the proliferation, migration, invasion, and EMT of HCCLM3 cells in vitro (Fig. 3a–f, *P* < 0.05). In addition, the treatment with BRD9 inhibitor (I-BRD9) suppressed the proliferation, migration, invasion, and EMT of HCCLM3 cells (Supplementary Fig. 5A–F, *P* < 0.05). Collectively, our results reveal that BRD9 promotes the malignant behaviors of HCC cells.

### BRD9 knockdown suppresses the growth and lung metastasis of HCC cells in vivo

**Fig. 4:**
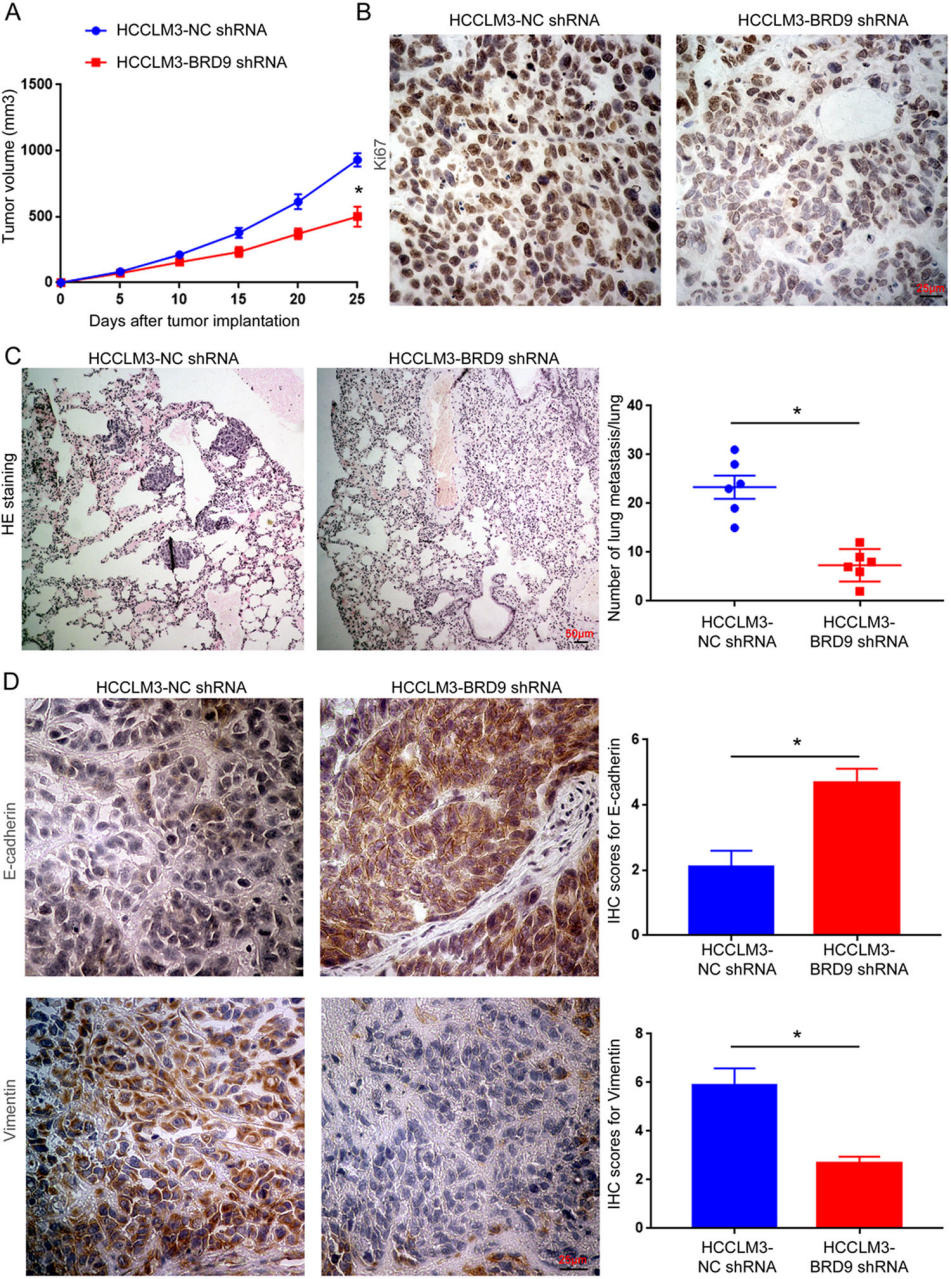
BRD9 knockdown suppresses the growth and metastasis of HCC in vivo. **a** The subcutaneous tumor formation model was established to evaluate the effect of BRD9 knockdown on HCC growth in nude mice. Tumor volumes were measured every 5 days after subcutaneous injection and compared between HCCLM3 cells with BRD9 knockdown (HCCLM3-BRD9 shRNA) or negative control HCCLM3 cells (HCCLM3-NC shRNA). *n* = 6, *P < 0.05 by ANOVA. **b** The tumors formed by HCCLM3-BRD9 shRNA and HCCLM3-NC shRNA cells were subjected to immunohistochemical staining for the proliferation marker Ki67. The percentages of Ki67-positive cells were compared between the two groups. Scale bar: 25 μm. *n* = five randomly selected fields of six samples, *P < 0.05 by *t*-test. **c** A tail-vein injection model was used to evaluate the effect of BRD9 knockdown on the metastasis of HCCLM3 cells in vivo. H&E staining was performed to identify the metastatic nodules of HCC cells in the lungs. Scale bar: 50 μm. *n* = 6, *P < 0.05 by *t*-test. **d** The tumors formed by HCCLM3-BRD9 shRNA and HCCLM3-NC shRNA cells were subjected to immunohistochemical staining for EMT markers, including E-cadherin and vimentin. IHC scores for E-cadherin and vimentin were compared between the two groups. Scale bar: 25 μm. *n* = five randomly selected fields of six samples, *P < 0.05 by *t*-test.

To investigate the influence of BRD9 on the growth of HCC cells in vivo, HCCLM3 cells with a BRD9 knockdown (HCCLM3-BRD9 shRNA) or the negative control group (HCCLM3-NC shRNA) were injected subcutaneously into nude mice. As shown in Fig. 4a, the tumor volume in the BRD9 knockdown group was significantly decreased compared with that in the control group (*P* < 0.05). The results of Ki67 staining demonstrated that tumor tissues from the BRD9 knockdown group exhibited a decreased percentage of Ki67-positive cells than those from the control group (Fig. 4b and Supplementary Fig. 6, *P* < 0.05). The effect of BRD9 on the metastatic ability of HCC cells was evaluated using a tail-vein injection model. The knockdown of BRD9 inhibited the lung metastasis of HCCLM3 cells, as suggested by fewer metastatic nodules formed by the HCCLM3 cells with the BRD9 knockdown in the lungs of the nude mice (Fig. 4c, *P* < 0.05). Notably, subcutaneous tumor tissues were subjected to IHC staining for E-cadherin and vimentin. Tumor tissues in the HCCLM3-BRD9 shRNA group showed an increased level of E-cadherin and a decreased level of vimentin (Fig. 4d, *P* < 0.05). Taken together, these data suggest that the BRD9 knockdown represses the growth and lung metastasis of HCC cells in vivo.

### BRD9 activates the TUFT1/AKT pathway in HCC cells

**Fig. 5:**
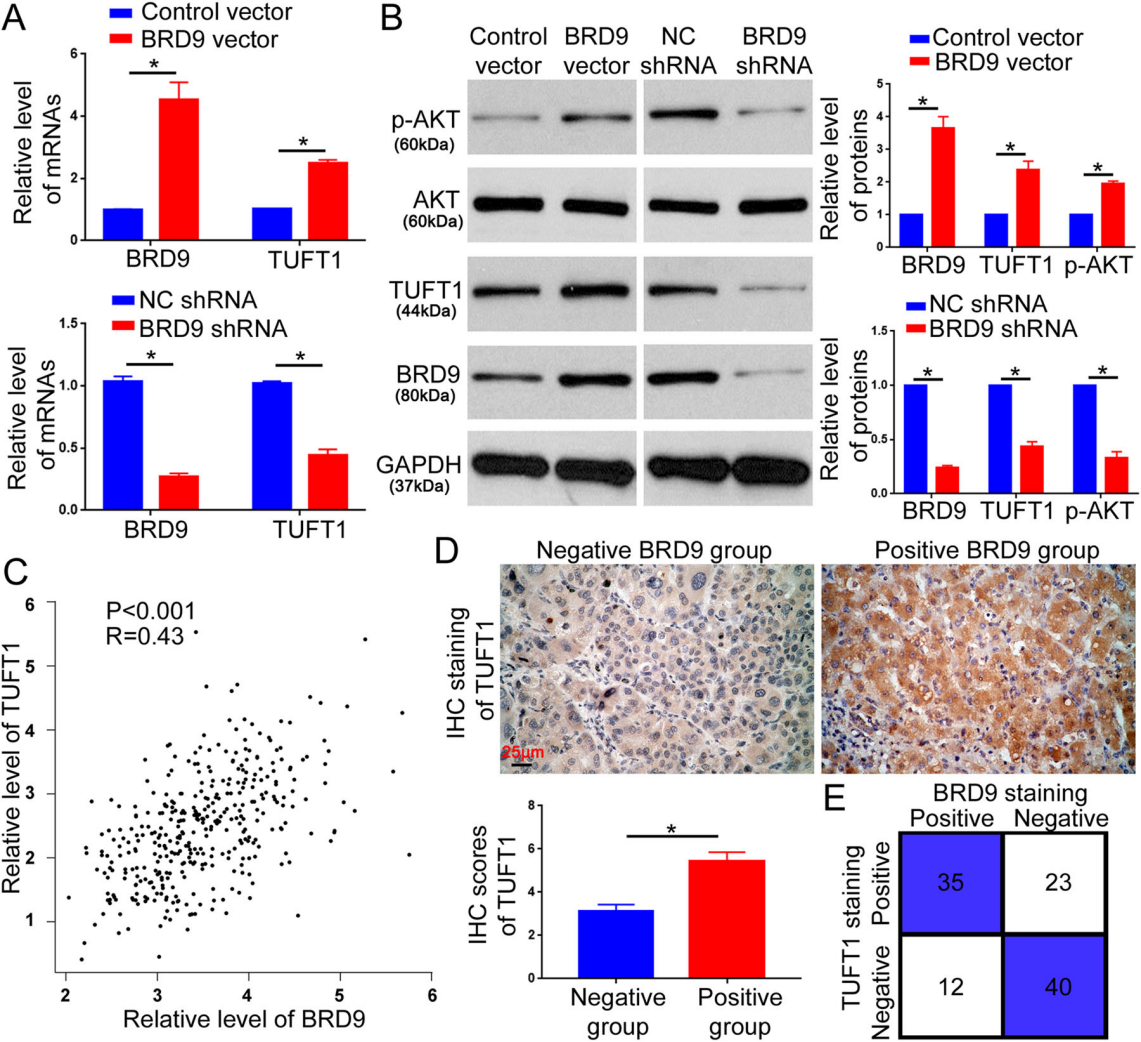
BRD9 upregulates TUFT1 expression in HCC. **a** qRT-PCR was performed to determine the regulatory effect of BRD9 overexpression or knockdown on the TUFT1 level in Hep3B or HCCLM3 cells. *n* = three independent experiments, *P < 0.05 by *t*-test. **b** Western blotting was performed to determine the regulatory effect of BRD9 overexpression or knockdown on the TUFT1/AKT pathway in Hep3B or HCCLM3 cells. *n* = three independent experiments, *P < 0.05 by *t*-test. **c** The TCGA database was explored to evaluate the correlation between BRD9 and TUFT1 levels in HCC tissues. *n* = 374, *P < 0.05 by Spearman’s rank correlation test. **d** IHC was performed to evaluate the correlation between BRD9 protein and TUFT1 protein in the HCC tissues. Scale bar: 25 μm. *n* = 110, *P < 0.05 by *t*-test. **e** The positive rate of TUFT1 staining was compared between HCC tissues with positive and negative BRD9 staining. *P < 0.05 by Pearson *χ*^2^ test.

Our previous study demonstrated that TUFT1 was a critical oncogene in HCC and facilitated tumor growth and metastasis by activating the AKT pathway^16^. Therefore, in this study, we explored whether BRD9 exerted its oncogenic function in HCC cells by modulating the TUFT1/AKT pathway. To this end, qRT-PCR and western blotting were performed to evaluate the effect of BRD9 on the expression of TUFT1. The data revealed that the overexpression of BRD9 increased the mRNA and protein levels of TUFT1 in Hep3B cells, whereas the BRD9 knockdown reduced TUFT1 expression in HCCLM3 cells (*P* < 0.05, Fig. 5a, b). Accordingly, BRD9 overexpression increased p-AKT, whereas its knockdown decreased p-AKT in HCC cells (*P* < 0.05, Fig. 5b). To further validate the regulatory effect of BRD9 on TUFT1, we explored the TCGA database for HCC and found that the level of BRD9 mRNA showed a remarkable positive correlation with the expression of TUFT1 mRNA in HCC tissues (Fig. 5c, *P* < 0.05). IHC staining demonstrated that the expression of TUFT1 protein in HCC tissues with positive BRD9 staining was significantly higher than that in tissues with negative BRD9 staining (Fig. 5d, *P* < 0.05). In addition, compared with tissues with negative BRD9 staining, HCC tissues with positive BRD9 staining showed an increased positive rate of TUFT1 (35/47 vs 23/63, Fig. 5e). Taken together, these data indicate that BRD9 upregulates the expression of TUFT1 and activates AKT signaling in HCC cells.

### BRD9 epigenetically regulates TUFT1 via P300

**Fig. 6:**
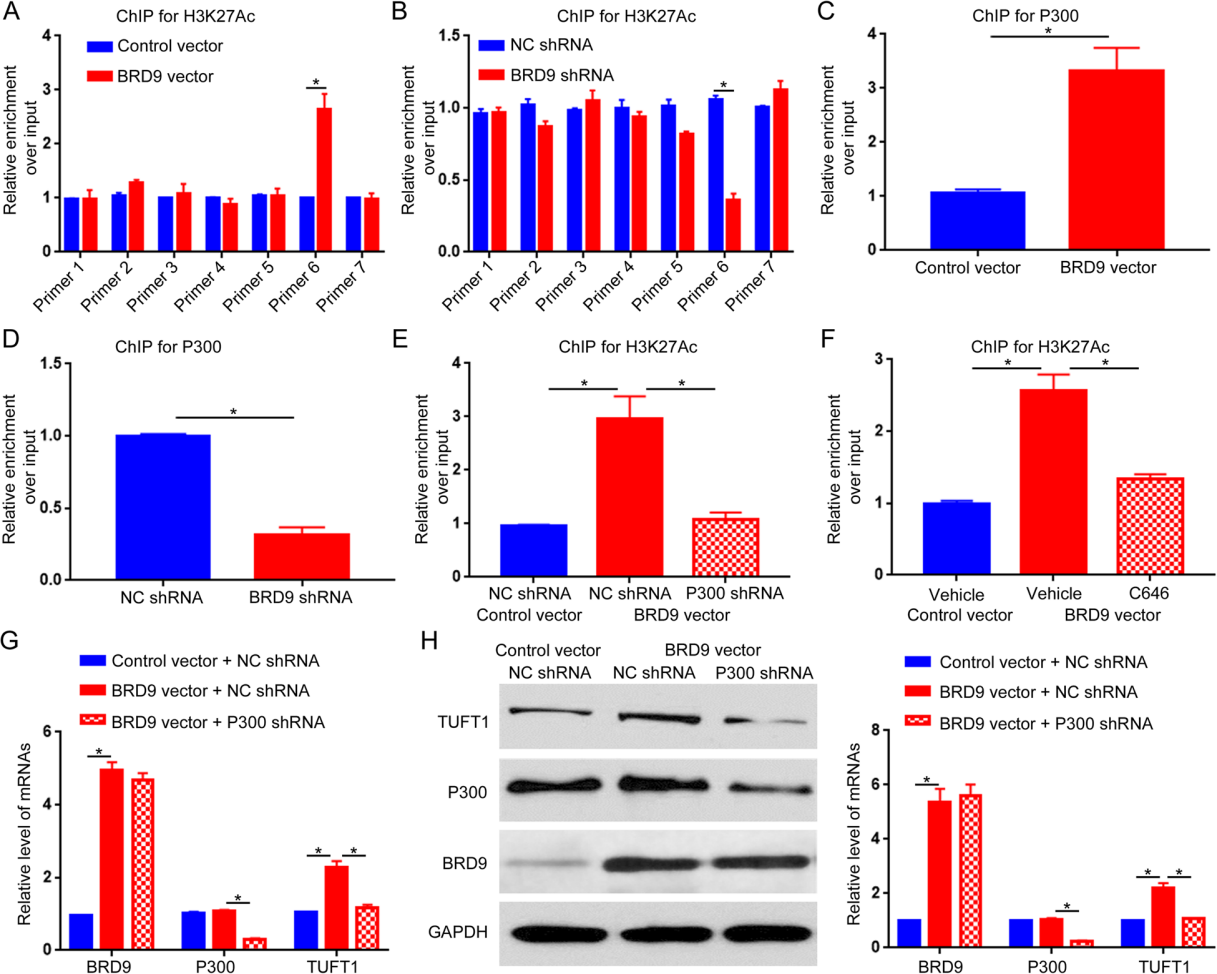
BRD9 epigenetically regulates TUFT1 via P300 acetyltransferase. **a** The ChIP assay was performed to determine the influence of BRD9 overexpression on H3K27Ac of the TUFT1 promoter in Hep3B cells. *n* = three independent experiments, *P < 0.05 by *t*-test. **b** The ChIP assay was performed to determine the influence of BRD9 knockdown on H3K27Ac of the TUFT1 promoter in HCCLM3 cells. *n* = three independent experiments, *P < 0.05 by *t*-test. **c** The ChIP assay was performed to determine the influence of BRD9 overexpression on P300 binding to the TUFT1 promoter in Hep3B cells. *n* = three independent experiments, *P < 0.05 by *t*-test. **d** The ChIP assay was performed to determine the influence of BRD9 knockdown on P300 binding to the TUFT1 promoter in HCCLM3 cells. *n* = three independent experiments, *P < 0.05 by *t*-test. **e** The ChIP assay was performed to examine the influence of P300 knockdown on H3K27Ac in the TUFT1 promoter after overexpressing BRD9 in Hep3B cells. *n* = three independent experiments, *P < 0.05 by *t*-test. **f** The ChIP assay was performed to examine the influence of C646 treatment on H3K27Ac in the TUFT1 promoter after overexpressing BRD9 in Hep3B cells. *n* = three independent experiments, *P < 0.05 by *t*-test. **g** qRT-PCR and **h** western blotting were performed to evaluate the influence of P300 knockdown on TUFT1 expression after overexpressing BRD9 in Hep3B cells. *n* = three independent experiments, *P < 0.05 by *t*-test.

Previous studies confirmed that BRD9 could affect gene transcription by regulating the epigenetic status (H3K27Ac and H3K4Me3) of targeted genes. To determine whether BRD9 affected the epigenetic status of the TUFT1 gene, we performed ChIP for BRD9, H3K27Ac, H3K14Ac, H3K9Ac, and H3K4Me3 using walking primers along with TUFT1 promoter (Supplementary Fig. 7A). ChIP for BRD9 revealed that BRD9 could bind to the promoter region of TUFT1 covered by the No. 6 primer (Supplementary Fig. 7B). ChIP for H3K14Ac, H3K9Ac, and H3K4Me3 failed to demonstrate any obvious change for these markers along with TUFT1 promoter after BRD9 overexpression or knockdown (Supplementary Fig. 8A–F). Interestingly, ChIP for H3K27Ac revealed that BRD9 overexpression increased H3K27Ac in the TUFT1 promoter region with the No. 6 primer (Fig. 6a, *P* < 0.05), whereas BRD9 knockdown decreased H3K27Ac in that region (Fig. 6b, *P* < 0.05). As BRD9 lacks histone acetyltransferase (HAT) activity^6,7^, we further investigated the potential mechanism by which BRD9 affected H3K27Ac in the TUFT1 promoter region. In our previous study, P300, a well-recognized epigenetic regulator, was found to regulate H3K27Ac in the promoter region of cancer-associated genes^17,23,24^. Thus, we explored whether P300 was involved in the epigenetic regulation of BRD9 on the TUFT1 promoter. The ChIP assay for P300 demonstrated that BRD9 overexpression increased P300 engagement with the TUFT1 promoter (Fig. 6c, *P* < 0.05). The knockdown of BRD9 decreased P300 binding to the TUFT1 promoter (Fig. 6d, *P* < 0.05). These results indicate that BRD9 can facilitate the engagement of P300 to the promoter region of TUFT1. Further, we validated that the engagement of P300 in the TUFT1 promoter was critical for the upregulation of H3K27Ac and TUFT1 expression induced by BRD9 overexpression. The ChIP assay showed that knockdown of P300 abrogated the increase in H3K27Ac in the TUFT1 promoter induced by BRD9 overexpression (Fig. 6e, *P* < 0.05). Treatment of C646, an inhibitor of P300, also inhibited H3K27Ac in the TUFT1 promoter induced by BRD9 overexpression (Fig. 6f, *P* < 0.05). The qRT-PCR and western blotting confirmed that the knockdown of P300 prevented the upregulation of TUFT1 induced by BRD9 overexpression (Fig. 6g, h, *P* < 0.05). These results suggest that BRD9 epigenetically turns on TUFT1 expression by facilitating the binding of P300 to the TUFT1 promoter and increasing H3K27Ac in the promoter region.

### Activation of the TUFT1/AKT pathway mediates the oncogenic role of BRD9 in HCC cells

**Fig. 7:**
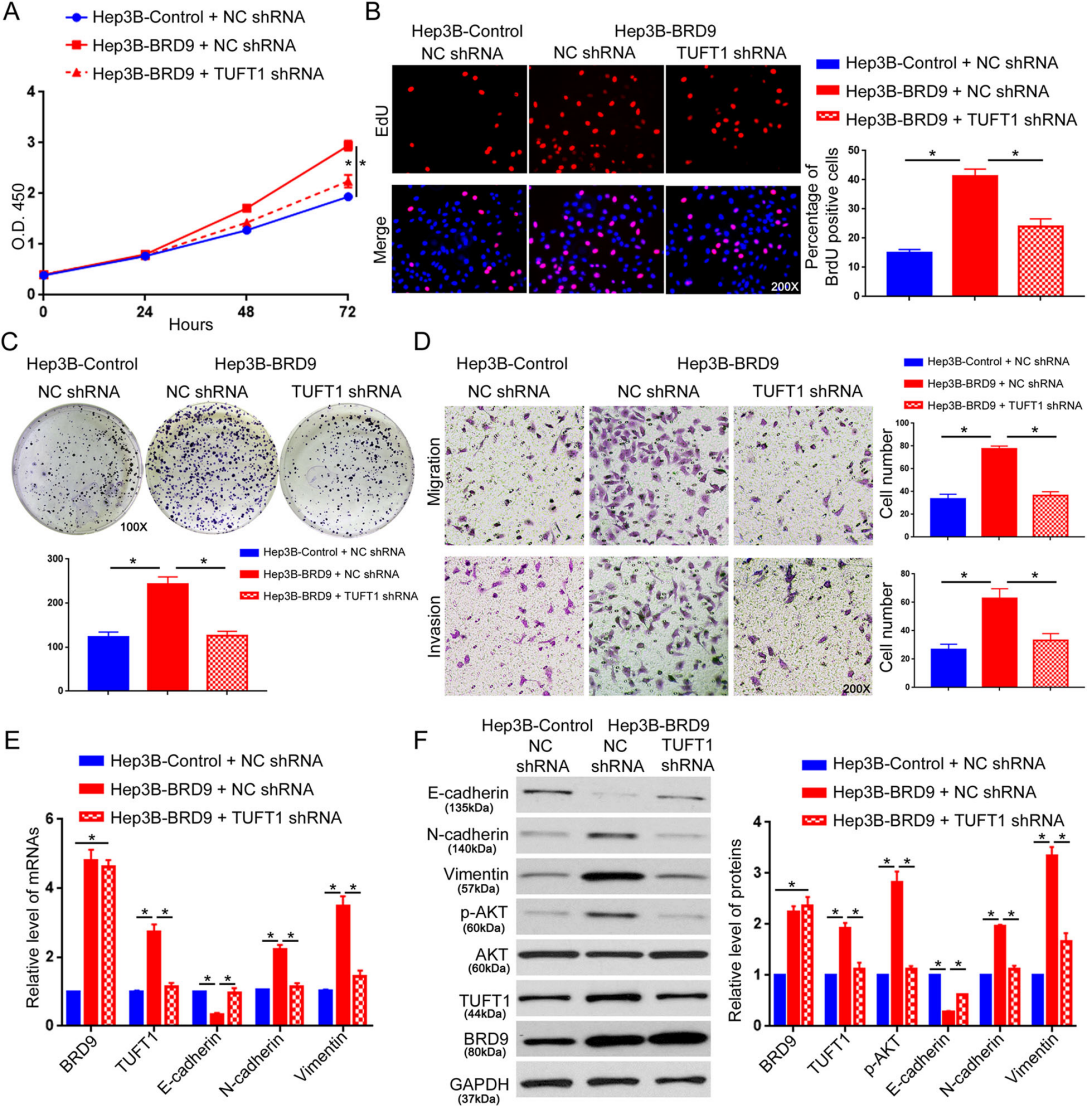
TUFT1 knockdown reverses the promoting effects of BRD9 on Hep3B cell growth and metastasis. Hep3B-control or Hep3B-BRD9 cells were transfected with TUFT1 shRNA or NC shRNA. **a** The MTT assay, **b** EdU assay, **c** colony formation assay, and **d** transwell assay were performed to examine the effect of TUFT1 knockdown on cell viability, proliferation, growth, migration, and invasion of Hep3B cells overexpressing BRD9. *n* = three independent experiments, *P < 0.05 by *t*-test or ANOVA. **e** qRT-PCR and **f** western blotting were performed to determine the effect of TUFT1 knockdown on the expression of EMT markers (E-cadherin, N-cadherin, and vimentin) and AKT phosphorylation in Hep3B cells overexpressing BRD9. *n* = three independent experiments, *P < 0.05 by *t*-test.

**Fig. 8:**
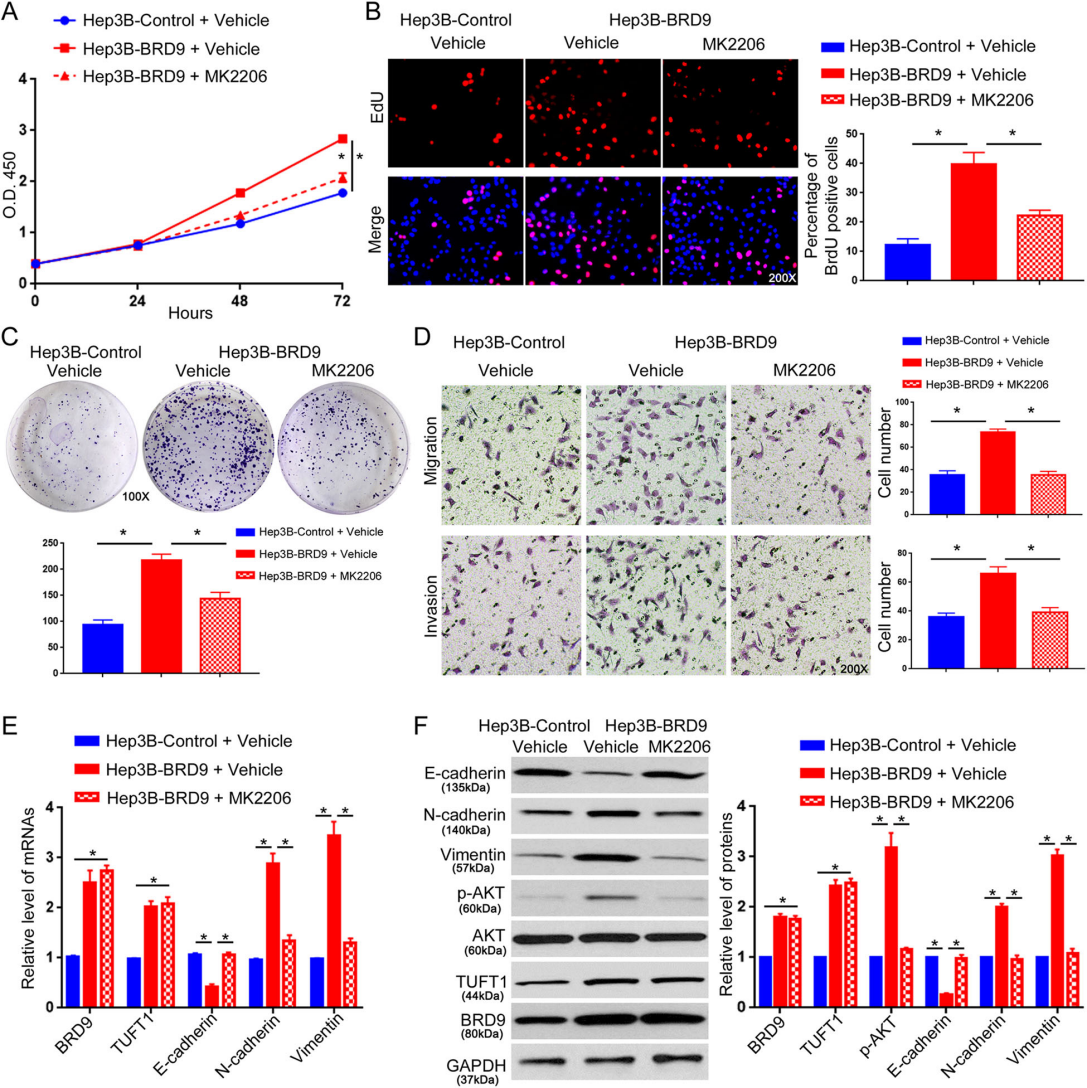
An AKT inhibitor abrogates the promoting effects of BRD9 on Hep3B cell growth and metastasis. Hep3B-control or Hep3B-BRD9 cells were treated with an AKT pathway inhibitor (MK2206) or vehicle. **a** The MTT assay, **b** EdU assay, **c** colony formation assay, and **d** Transwell assay were performed to examine the effect of MK2206 treatment on cell viability, proliferation, growth, migration, and invasion of Hep3B cells overexpressing BRD9. *n* = three independent experiments, *P < 0.05 by *t*-test or ANOVA. **e** qRT-PCR and **f** western blotting were performed to determine the effect of MK2206 treatment on the expression of EMT markers (E-cadherin, N-cadherin, and vimentin) and AKT phosphorylation in Hep3B cells overexpressing BRD9. *n* = three independent experiments, *P < 0.05 by *t*-test.

Last, we investigated whether the TUFT1/AKT pathway was critical for the oncogenic role of BRD9 in HCC. Knockdown of TUFT1 effectively blocked the increase in cell viability, proliferation, and colony formation that resulted from BRD9 overexpression in Hep3B cells (Fig. 7a–c, *P* < 0.05). The Transwell assay showed that the increase in cell migration and invasion induced by BRD9 overexpression was suppressed by TUFT1 knockdown (Fig. 7d, *P* < 0.05). Moreover, the decrease in E-cadherin and the increase in N-cadherin and vimentin induced by BRD9 overexpression was reversed by TUFT1 knockdown in Hep3B cells (Fig. 7e, f, *P* < 0.05). Consistently, treatment with MK2206, an inhibitor of AKT phosphorylation, effectively blocked the enhancement of proliferation, migration, invasion, and EMT in Hep3B cells induced by BRD9 overexpression (Fig. 8a–f, *P* < 0.05). Moreover, the forced expression of TUFT1 reversed the inhibition of proliferation, migration, invasion, and EMT of HCCLM3 cells that resulted from BRD9 knockdown (Supplementary Fig. 9A–F, *P* < 0.05). Taken together, these data demonstrate that BRD9 exerts its promoting effects on the growth and metastasis of HCC cells by activating the TUFT1/AKT pathway.

## Discussion

In this study, we identify BRD9 as a key oncogenic regulator of HCC growth and metastasis and reveal the mechanism underlying the biological functions of BRD9 in HCC. We demonstrated that the level of BRD9 was upregulated in HCC tissues by analyzing data from publicly available databases and measuring the expression level of BRD9 using qRT-PCR, western blotting, and IHC in the resected HCC tissues. The elevated level of BRD9 in HCC tissues was correlated with poor clinicopathological features and an unfavorable prognosis in HCC patients.

BRD9 has been confirmed to be a component of the SWI-SNF complex and is involved in chromatin remodeling and transcriptional regulation^9^. BRD9 engagement mainly occurs at the enhancer and promoter level in a cell type-specific manner and thus regulates various cellular functions^7,25^. A study by Hohmann et al. ^8^ showed that BRD9 was required for the proliferation of AML cells by promoting MYC transcription. In MCF-10A cells expressing both oncogenic KRAS and PIK3CA, BRD9 was observed to bind at the TSS of the vimentin gene and enhanced the expression of vimentin^11^. In our study, BRD9 overexpression promoted the proliferation, migration, invasion, and EMT of Hep3B cells. In contrast, BRD9 knockdown suppressed these biological functions in HCCLM3 cells. The effects of BRD9 on cell growth and metastasis were further validated by the subcutaneous injection model and tail-vein injection model in nude mice. In HCC cells, we also tested whether BRD9 affected MYC expression and failed to observe an obvious alteration in MYC expression after BRD9 knockdown (data not shown). Different from the direct binding of BRD9 on the vimentin gene in MCF-10A cells, ChIP for BRD9 failed to show the direct binding of BRD9 to the promoter regions of E-cadherin, N-cadherin, and vimentin (data not shown). These results indicate that the regulatory mechanisms of BRD9 on cell growth and metastasis varied between different cancer types. Owing to its cell type-specific manner of chromatin targeting, the biological functions of BRD9 in other human cancer cells requires further investigation.

The sustained proliferative ability and enhanced metastatic behavior are important hallmarks for cancer cells^26^. Activation of classical signaling pathways, including the PI3K/AKT pathway, ERK pathway, JNK pathway, Hedgehog pathway, and TGF-β pathway, has been observed in cancer cells to mediate cancer growth and metastasis^27–31^. Our previous study demonstrated that TUFT1 was overexpressed in HCC and promoted the proliferation, migration, invasion, and EMT of cancer cells by activating the AKT pathway^16^. Therefore, in this study, we explored whether the activation of TUFT1/AKT was associated with the oncogenic functions of BRD9 in HCC. Gain- and loss-of-function assays demonstrated that the expression of TUFT1 was under the regulation of BRD9 in HCC cells. This regulatory relationship was further supported by the positive correlation between TUFT1 and BRD9 expression levels in HCC tissues. Suppression of the TUFT1/AKT pathway in HCC cells using either TUFT1 shRNA or the AKT phosphorylation inhibitor (MK2206) abrogated the promoting effects of BRD9 on cell growth, metastasis, and EMT. These data reveal that BRD9 exerts oncogenic effects in HCC by activating the TUFT1/AKT pathway.

Epigenetic alteration and chromatin remodeling have been widely recognized to be responsible for the aberrant expression of cancer-associated genes^32,33^. Previous studies indicate that BRD9 is a subunit of SWI-SNF complexes and is associated with epigenetic changes in cancer cells^8–10,13^. Dysregulation of the bromodomain-containing protein BRD9 was coincident with altered histone acetylation (K9/K14/K27 of H3) in HHi (Hedgehog inhibitors)-resistant BCC (basal cell carcinoma)^12^. Therefore, this study investigated whether BRD9 affected the epigenetic status in the TUFT1 promoter. ChIP assays demonstrated that H3K27Ac in the TUFT1 promoter region was increased upon BRD9 overexpression and decreased upon BRD9 knockdown. We failed to observe the changes of other epigenetic markers (H3K14Ac, H3K9Ac, or H3K4Me3) around the TUFT1 promoter regions after BRD9 overexpression or knockdown. These results indicate that BRD9 could potentiate H3K27Ac in the TUFT1 promoter region. Different from other BRD-containing proteins (P300, PCAF, CREBBP) that harbor the HAT domain, BRD9 cannot regulate histone acetylation directly^6^. Our previous study demonstrated that P300 was able to affect the H3K27Ac of various cancer-related genes^17,24^. P300 was confirmed to be overexpressed in HCC and promoted the progression of HCC^34–36^. The ChIP assay for P300 in our study demonstrated that the engagement of P300 in the TUFT1 promoter region was increased after BRD9 overexpression. In contrast, BRD9 knockdown reduced P300 engagement with the TUFT1 promoter. Western blotting revealed that the level of P300 protein was not affected upon BRD9 overexpression. Thus, this result indicates that BRD9 can facilitate the engagement of P300 with the TUFT1 promoter. Furthermore, the increase in H3K27Ac in the TUFT1 promoter induced by BRD9 was abrogated by P300 inhibition (P300 shRNA and C646 treatment). Accordingly, the elevation of TUFT1 expression that resulted from BRD9 overexpression was also suppressed by P300 inhibition. Taken together, these data indicate that BRD9 can facilitate the binding of P300 to the TUFT1 promoter, which resulted in increased H3K27Ac, and thus leads to the increased expression level of TUFT1 in HCC cells. However, the exact mechanism by which BRD9 facilitated the interaction between P300 and TUFT1’s promoter remains to be elucidated in the future.

In summary, this study demonstrates that BRD9 is an oncogenic protein in HCC and enhances the proliferation, metastasis, and EMT of HCC cells. The elevated expression of BRD9 in HCC tissues is associated with poor clinicopathological features and an unfavorable prognosis. Furthermore, we reveal that BRD9 facilitates the binding of P300 to the TUFT1 promoter, and epigenetically turns on the expression of TUFT1 by increasing H3K27Ac in the TUFT1 promoter. Activation of the TUFT1/AKT pathway is required for the promoting effects of BRD9 on the growth and metastasis of HCC cells. This study demonstrates that BRD9 may represent a novel biomarker for HCC and serve as a potential therapeutic target for HCC treatment.

## Supplementary information

Supplementary Table 1

Supplementary Table 2

Supplementary Table 3

Supplementary Figure Legends

Supplementary Figure 1

Supplementary Figure 2

Supplementary Figure 3

Supplementary Figure 4

Supplementary Figure 5

Supplementary Figure 6

Supplementary Figure 7

Supplementary Figure 8

Supplementary Figure 9
