# Supplementary material for: Bromodomain-containing protein 9 promotes the growth and metastasis of human hepatocellular carcinoma by activating the TUFT1/AKT pathway
Source: Cell Death Dis. 2020 Sep 9;11(9):730. doi: 10.1038/s41419-020-02943-7 (PMC7481201; doi:10.1038/s41419-020-02943-7)
Supplement: Supplementary file 1 — Supplementary Table 1 [file 41419_2020_2943_MOESM1_ESM.doc]

**Supplementary Table 1.** Correlation between the clinicopathologic characteristics and BRD9 expression in hepatocellular carcinoma

| **Clinical features** | | **No. of patients** | **IHC staining for BRD9** | | ***P*** |
| --- | --- | --- | --- | --- | --- |
|  |  |  | **Negative** | **Positive** |  |
| Age | <50 | 42 | 26 | 16 | 0.440 |
|  | ≥50 | 68 | 37 | 31 |  |
| Gender | Male | 87 | 50 | 37 | 0.935 |
|  | Female | 23 | 13 | 10 |  |
| HBV infection | Absent | 31 | 20 | 11 | 0.336 |
|  | Present | 79 | 43 | 36 |  |
| Cirrhosis | Absent | 45 | 30 | 15 | 0.097 |
|  | Present | 65 | 33 | 32 |  |
| AFP level (ng/mL) | <20 | 37 | 25 | 12 | 0.120 |
|  | ≥20 | 73 | 38 | 35 |  |
| Tumor size (cm) | <5 | 36 | 26 | 10 | 0.027* |
|  | ≥5 | 74 | 37 | 37 |  |
| Venous infiltration | Absent | 60 | 42 | 18 | 0.003* |
|  | Present | 50 | 21 | 29 |  |
| TNM stage | I+II | 87 | 56 | 31 | 0.003* |
|  | III+IV | 23 | 7 | 16 |  |

HBV, hepatitis B virus; TNM, tumor-node-metastasis. * indicates P<0.05.
