# Supplementary material for: Bromodomain-containing protein 9 promotes the growth and metastasis of human hepatocellular carcinoma by activating the TUFT1/AKT pathway
Source: Cell Death Dis. 2020 Sep 9;11(9):730. doi: 10.1038/s41419-020-02943-7 (PMC7481201; doi:10.1038/s41419-020-02943-7)
Supplement: Supplementary file 2 — Supplementary Table 2 [file 41419_2020_2943_MOESM2_ESM.docx]

**Supplementary Table 2 The primer sequences used in this study**

| **Gene** | **Primer** | **Sequence (5′-3′)** |
| --- | --- | --- |
| BRD9 | forward | ATGTTCCATGAAGCCTCCAG |
|  | reverse | AGCTCCTTCTTCACCTTCCC |
| TUFT1 | forward | AGAGCCAGCAGCGGAAAGT |
|  | reverse | TTGACTGGATCACAGCTTTTGAA |
| E-cadherin | forward | GGGGTACCTGTCTCTCTACAAAAAGGCA |
|  | reverse | GGAAGATCTGGGCTGGAGCGGGCTGGAGT |
| N-cadherin | forward | GGGTGGAGGAGAAGAAGACCAG |
|  | reverse | GGCATCAGGCTCCACAGT |
| vimentin | forward | AGGAAATGGCTCGTCACCTTCGTGAATA |
|  | reverse | GGACTGTCGGTTGTTAAGAACTAGAGCT |
| 18S | forward | AAACGGTACCACATCCAAG |
|  | reverse | CCTCCAATGGATCCTCGTTA |
| Primer 1 | forward | CCTATACAAGGGGGCGGTTC |
|  | reverse | GGCGGACTTAGAAACCGGG |
| Primer 2 | forward | GTTAAAGCAGCCCCATGTCGC |
|  | reverse | GAACCGCCCCCTTGTATAGG |
| Primer 3 | forward | GGTGGCTTACGTATAGGGAGAG |
|  | reverse | GCGACATGGGGCTGCTTTAA |
| Primer 4 | forward | GGGCAGCGTCAGTAAAAGAG |
|  | reverse | CTCTCCCTATACGTAAGCCACC |
| Primer 5 | forward | AAGCCATCTGCCAAGAGCAG |
|  | reverse | CTCTTTTACTGACGCTGCCC |
| Primer 6 | forward | CCTAACTGACATAGGACCCAGTC |
|  | reverse | CTGCTCTTGGCAGATGGCTT |
| Primer 7 | forward | GACTGGGTCCTATGTCAGTTAGG |
|  | reverse | TCAGGCTGGTCTCAAACTCCT |
