# Supplementary material for: Bromodomain-containing protein 9 promotes the growth and metastasis of human hepatocellular carcinoma by activating the TUFT1/AKT pathway
Source: Cell Death Dis. 2020 Sep 9;11(9):730. doi: 10.1038/s41419-020-02943-7 (PMC7481201; doi:10.1038/s41419-020-02943-7)
Supplement: Supplementary file 3 — Supplementary Table 3 [file 41419_2020_2943_MOESM3_ESM.docx]

**Supplementary Table 3. The statistical analysis for quantifying BRD9 differential expression in TCGA database and GEO database.**

| **Database** | **TCGA** | |  | **GSE14323** | |  | **GSE14520** | |  | **GSE1898** | |  | **GSE32649** | |  | **GSE6764** | |
| --- | --- | --- | --- | --- | --- | --- | --- | --- | --- | --- | --- | --- | --- | --- | --- | --- | --- |
| **Tissue type** | **HCC** | **Non-tumor** |  | **HCC** | **Non-tumor** |  | **HCC** | **Non-tumor** |  | **HCC** | **Non-tumor** |  | **HCC** | **Non-tumor** |  | **HCC** | **Non-tumor** |
| **Number** | 369 | 50 |  | 38 | 77 |  | 225 | 220 |  | 22 | 21 |  | 99 | 86 |  | 35 | 23 |
| **Mean±SEM** | 0.173±0.030 | 0.019±0.007 |  | 1.782±0.036 | 1.555±0.025 |  | 1.078±0.040 | 0.734±0.028 |  | 1.765±0.082 | 1.155±0.046 |  | 0.050±0.011 | 0.010±0.005 |  | 3.068±0.078 | 2.577±0.054 |
| **Method** | Unpaired t test | |  | Unpaired t test | |  | Unpaired t test | |  | Unpaired t test | |  | Unpaired t test | |  | Unpaired t test | |
| **t value** | 5.095 | |  | 5.210 | |  | 7.037 | |  | 6.411 | |  | 4.553 | |  | 4.651 | |
| **P value** | <0.0001 | |  | <0.0001 | |  | <0.0001 | |  | <0.0001 | |  | <0.001 | |  | <0.001 | |
