# Supplementary material for: Bromodomain-containing protein 9 promotes the growth and metastasis of human hepatocellular carcinoma by activating the TUFT1/AKT pathway
Source: Cell Death Dis. 2020 Sep 9;11(9):730. doi: 10.1038/s41419-020-02943-7 (PMC7481201; doi:10.1038/s41419-020-02943-7)
Supplement: Supplementary file 4 — Supplementary Figure Legends [file 41419_2020_2943_MOESM4_ESM.docx]

**Supplementary Figure legends**

**Supplementary Figure 1. Data for BRD9 expression in HCC from online databases.** (A) The expression of BRD9 in 23 types of human cancer on the GEPIA website. Tumor tissues were labeled as red while the corresponding normal tissues were labeled in green. (B) Differential expression for BRD9 in HCC in TCGA database and GEO databases (GSE14323, GSE14520, GSE1898, GSE32649, and GSE6764). *P<0.05 by t-test.

**Supplementary Figure 2. The level of BRD9 protein is increased in HCC tissues.** IHC staining for BRD9 in HCC tissue and normal liver tissue on Human Protein Altas website.

**Supplementary Figure 3. Elevated BRD9 expression predicts poor prognosis of HCC patients in the TCGA database.** Overall survival and disease-free survival were compared between HCC patients with the high and low levels of BRD9 based on the data in the TCGA database. Dash lines represent the 95% confidence interval for the survival curves.

**Supplementary Figure 4. The transduction efficacy of viral vectors containing BRD9 vector or BRD9 shRNA in HCC cells.** (A) qRT-PCR and (B) western blot were performed for BRD9 expression in Hep3B cells transduced with viral vectors containing the BRD9 vector or control vector. n = three independent experiments, *P<0.05 by t-test. (C) qRT-PCR and (D) western blot were performed for BRD9 expression in HCCLM3 cells transduced with viral vectors containing BRD9 shRNA or NC shRNA. n = three independent experiments, *P<0.05 by t-test.

**Supplementary Figure 5. BRD9 inhibitor (I-BRD9) suppresses the proliferation, migration, invasion, and EMT of HCCLM3 cells.** (A) MTT assay was performed to evaluate the effect of BRD9 inhibitor (I-BRD9) on the viability of HCCLM3 cells. n = three independent experiments, *P<0.05 by ANOVA. (B) EdU assay was performed to evaluate the effect of BRD9 inhibitor (I-BRD9) on the proliferation of HCCLM3 cells. n = five randomly selected fields of three independent experiments, *P<0.05 by t-test. (C) Colony formation assay was performed to evaluate the effect of BRD9 inhibitor (I-BRD9) on the proliferation of HCCLM3 cells. n = three independent experiments, *P<0.05 by t-test. (D) Transwell assay was used to investigate the effect of BRD9 inhibitor (I-BRD9) on the migration and invasion of HCCLM3 cells. n = five randomly selected fields of three independent experiments, *P<0.05 by t-test. (E) qRT-PCR and (F) western blot were performed to evaluate the effect of BRD9 inhibitor (I-BRD9) on the expression of EMT markers (E-cadherin, N-cadherin, and Vimentin). n = three independent experiments, *P<0.05 by t-test.

**Supplementary Figure 6. BRD9 knockdown decreases the Ki-67 positive cells in subcutaneous tumors.** Ki67 staining was performed for subcutaneous tumors formed by HCCLM3-BRD9 shRNA cells or HCCLM3-NC shRNA cells. n = five randomly selected fields of five independent experiments, *P<0.05 by t-test.

**Supplementary Figure 7. BRD9 can bind to the TUFT1 promoter.** (A) Six pairs of walking primers were designed along the TUFT1 promoter. (B) ChIP assay was performed to evaluate whether BRD9 can bind to the TUFT1 promoter in HCCLM3 cells. n= three independent experiments, *P<0.05 by t-test.

**Supplementary Figure 8. BRD9 fails to affect H3K14Ac or H3K9Ac or H3K4Me3 in the regions of the TUFT1 promoter.** (A) ChIP assay for H3K14Ac was performed after overexpressing BRD9 in Hep3B cells. (B) ChIP assay for H3K14Ac was performed after the BRD9 knockdown in HCCLM3 cells. (C) ChIP assay for H3K9Ac was performed after overexpressing BRD9 in Hep3B cells. (D) ChIP assay for H3K9Ac was performed after BRD9 knockdown in HCCLM3 cells. (E) ChIP assay for H3K4Me3 was performed after overexpressing BRD9 in Hep3B cells. (F) ChIP assay for H3K4Me3 was performed after the BRD9 knockdown in HCCLM3 cells.

**Supplementary Figure 9. TUFT1 overexpression abrogates the inhibitory effects of BRD9 knockdown on the growth and metastasis of HCCLM3 cells.** HCCLM3-NC shRNA or HCCLM3-BRD9 shRNA cells were transfected with TUFT1 vector or control vector. (A) MTT assay, (B) EdU assay, (C) colony formation assay, and (D) transwell assay were performed to examine the effect of TUFT1 overexpression on cell viability, proliferation, growth, migration, and invasion of HCCLM3 cells with BRD9 knockdown. n = three independent experiments, *P<0.05 by t-test or ANOVA. (E) qRT-PCR and (F) western blot were performed to determine the effect of TUFT1 overexpression on the expression of EMT markers (E-cadherin, N-cadherin, and Vimentin) and AKT phosphorylation in HCCLM3 cells with BRD9 knockdown. n = three independent experiments, *P<0.05 by t-test.
