# Supplementary figures and images for: Bromodomain-containing protein 9 promotes the growth and metastasis of human hepatocellular carcinoma by activating the TUFT1/AKT pathway
Source: Cell Death Dis. 2020 Sep 9;11(9):730. doi: 10.1038/s41419-020-02943-7 (PMC7481201; doi:10.1038/s41419-020-02943-7)

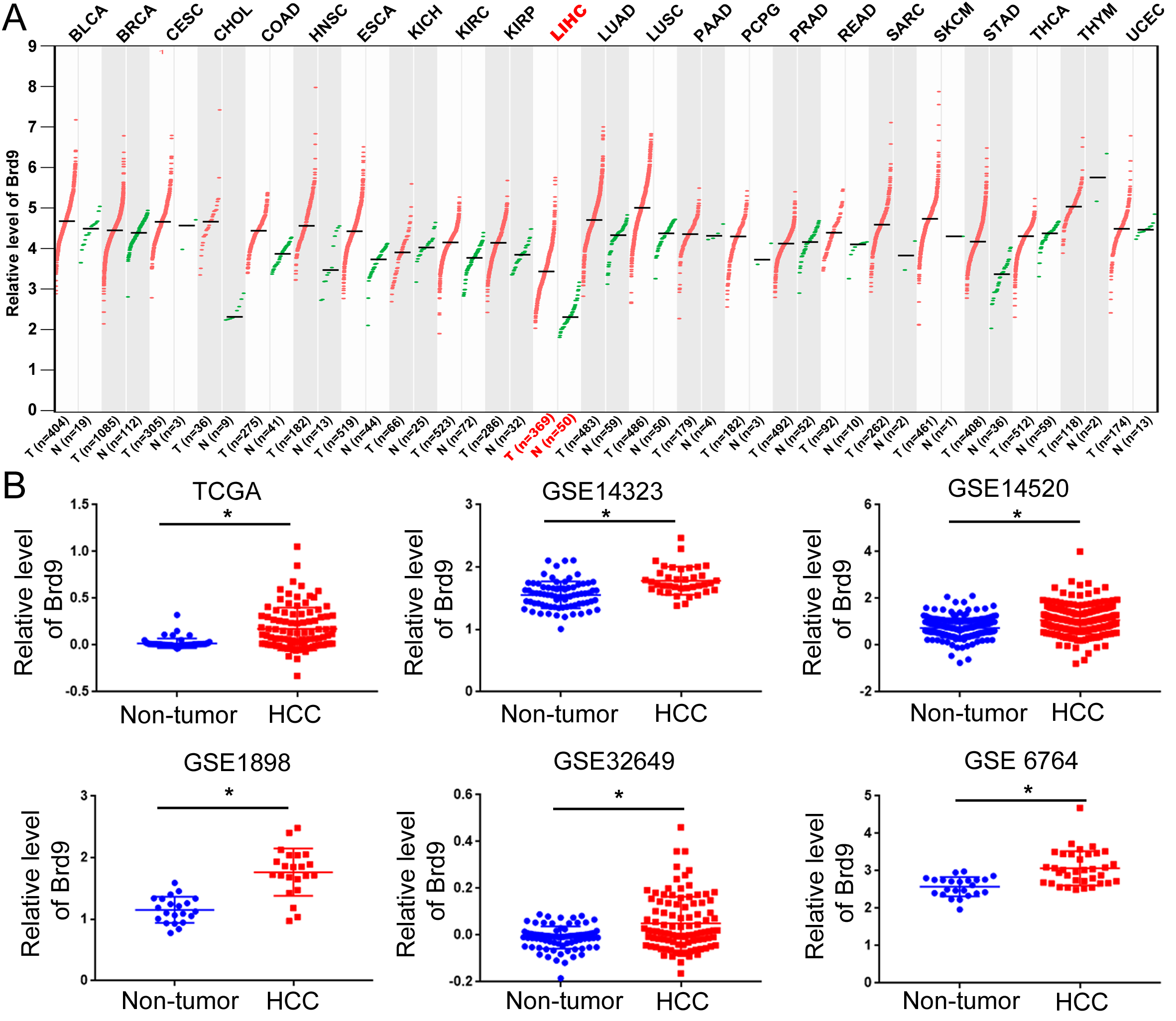

Supplement: Supplementary file 5 — Supplementary Figure 1 [file 41419_2020_2943_MOESM5_ESM.tif]

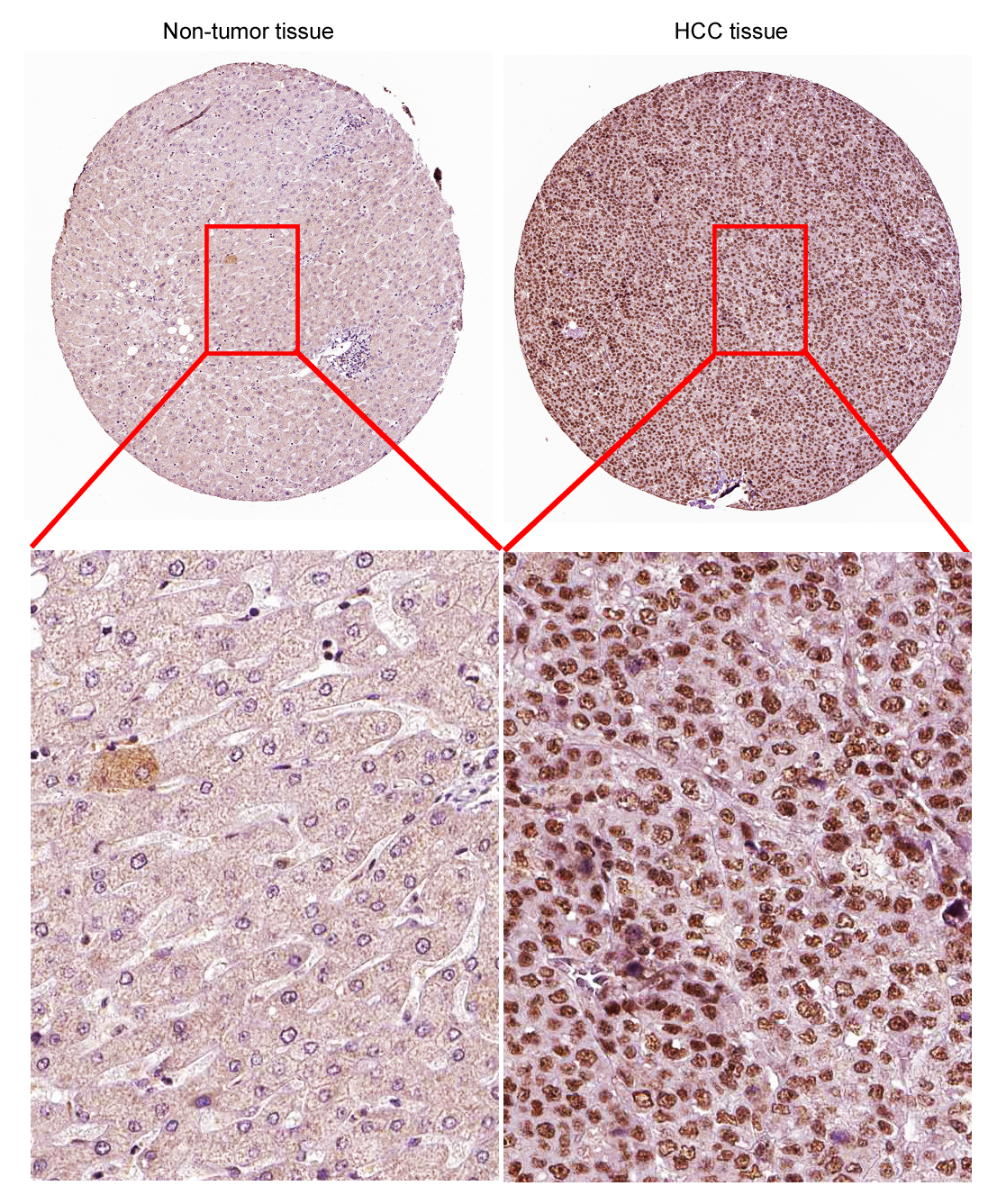

Supplement: Supplementary file 6 — Supplementary Figure 2 [file 41419_2020_2943_MOESM6_ESM.tif]

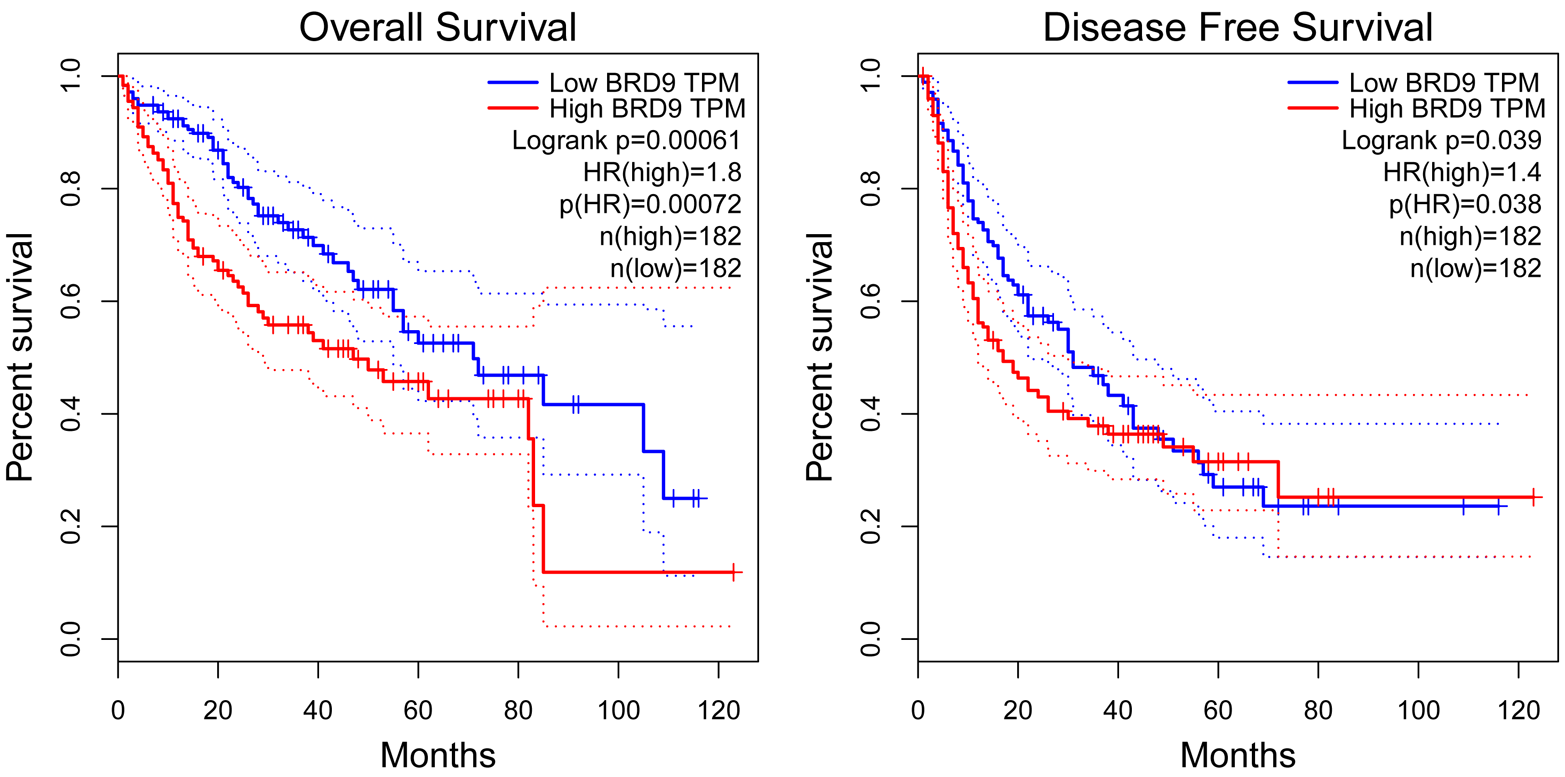

Supplement: Supplementary file 7 — Supplementary Figure 3 [file 41419_2020_2943_MOESM7_ESM.tif]

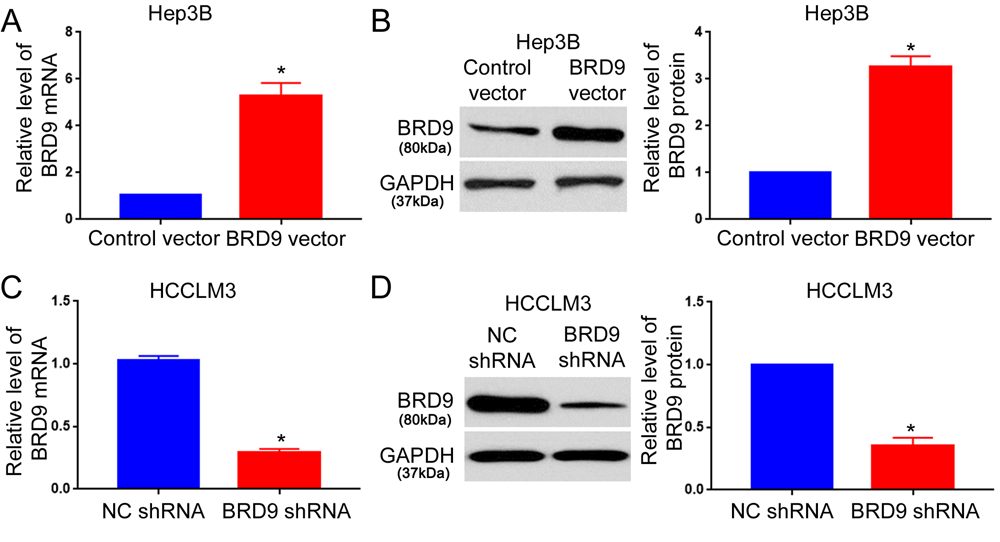

Supplement: Supplementary file 8 — Supplementary Figure 4 [file 41419_2020_2943_MOESM8_ESM.tif]

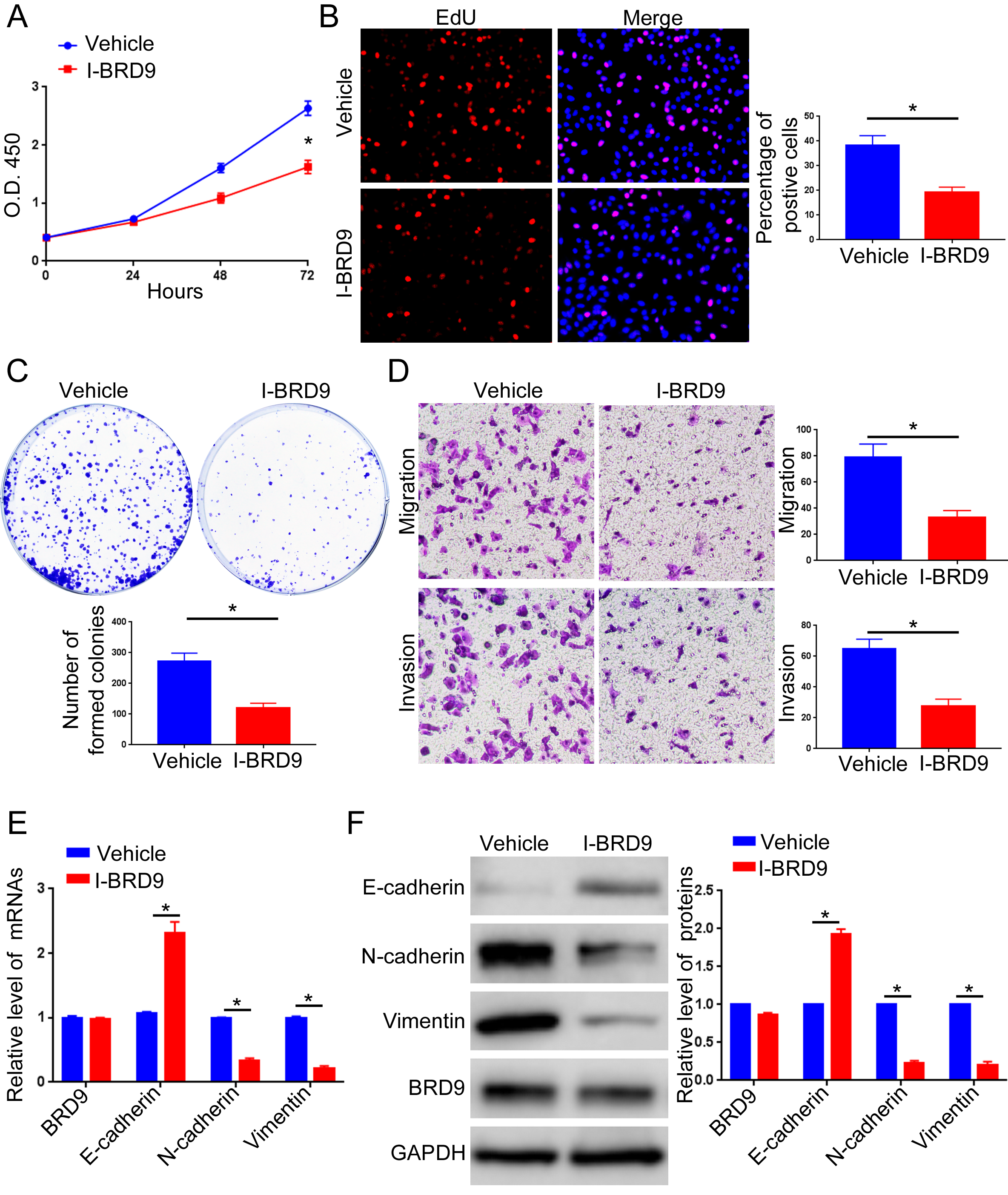

Supplement: Supplementary file 9 — Supplementary Figure 5 [file 41419_2020_2943_MOESM9_ESM.tif]

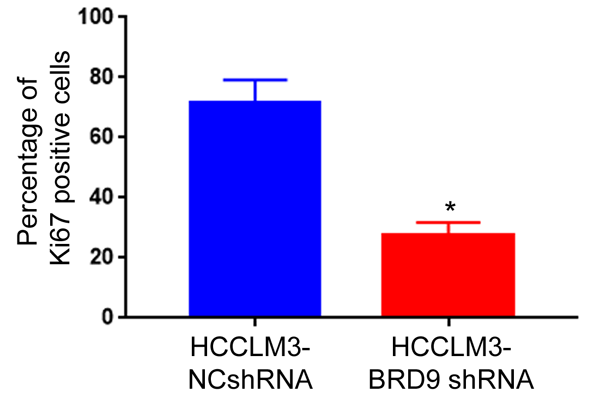

Supplement: Supplementary file 10 — Supplementary Figure 6 [file 41419_2020_2943_MOESM10_ESM.tif]

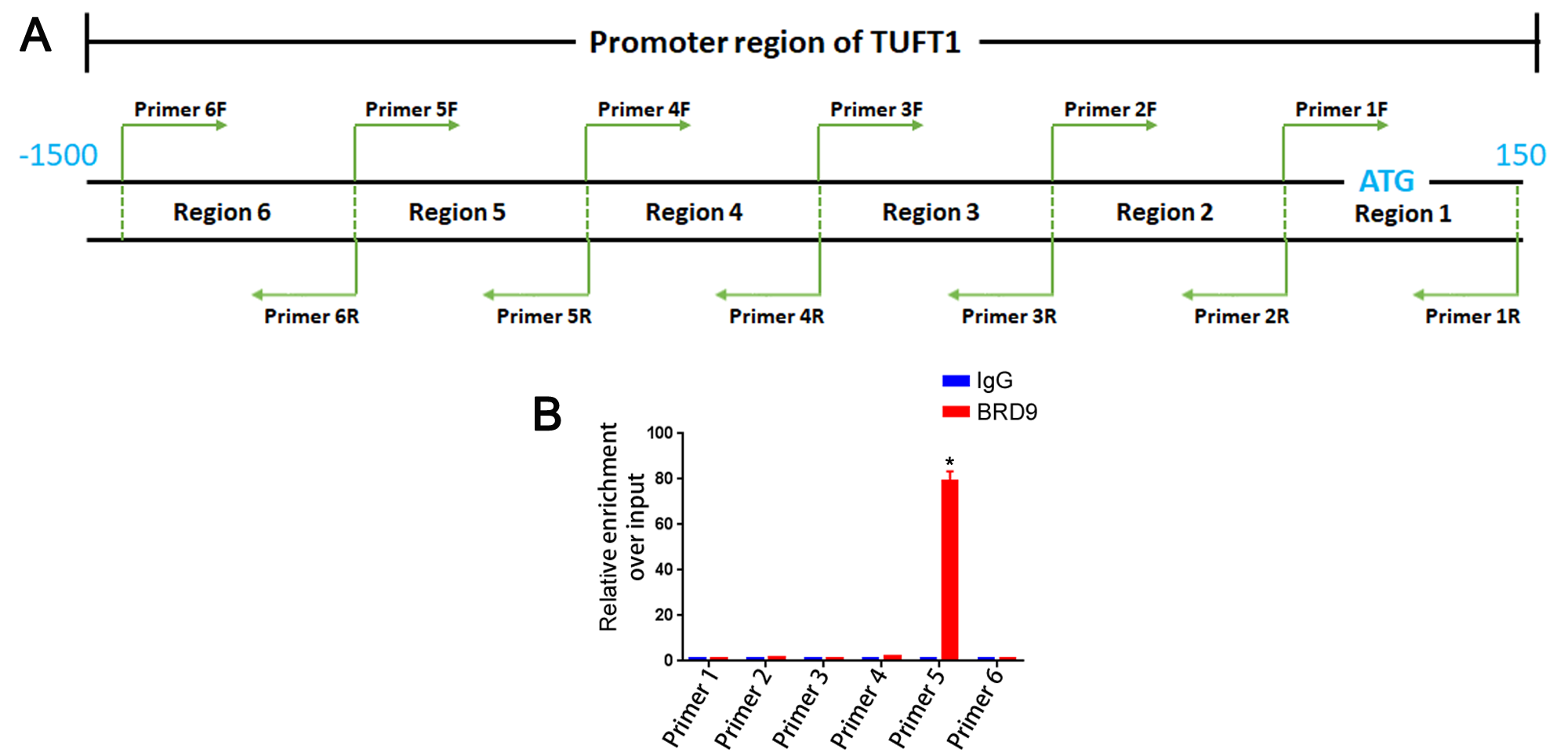

Supplement: Supplementary file 11 — Supplementary Figure 7 [file 41419_2020_2943_MOESM11_ESM.tif]

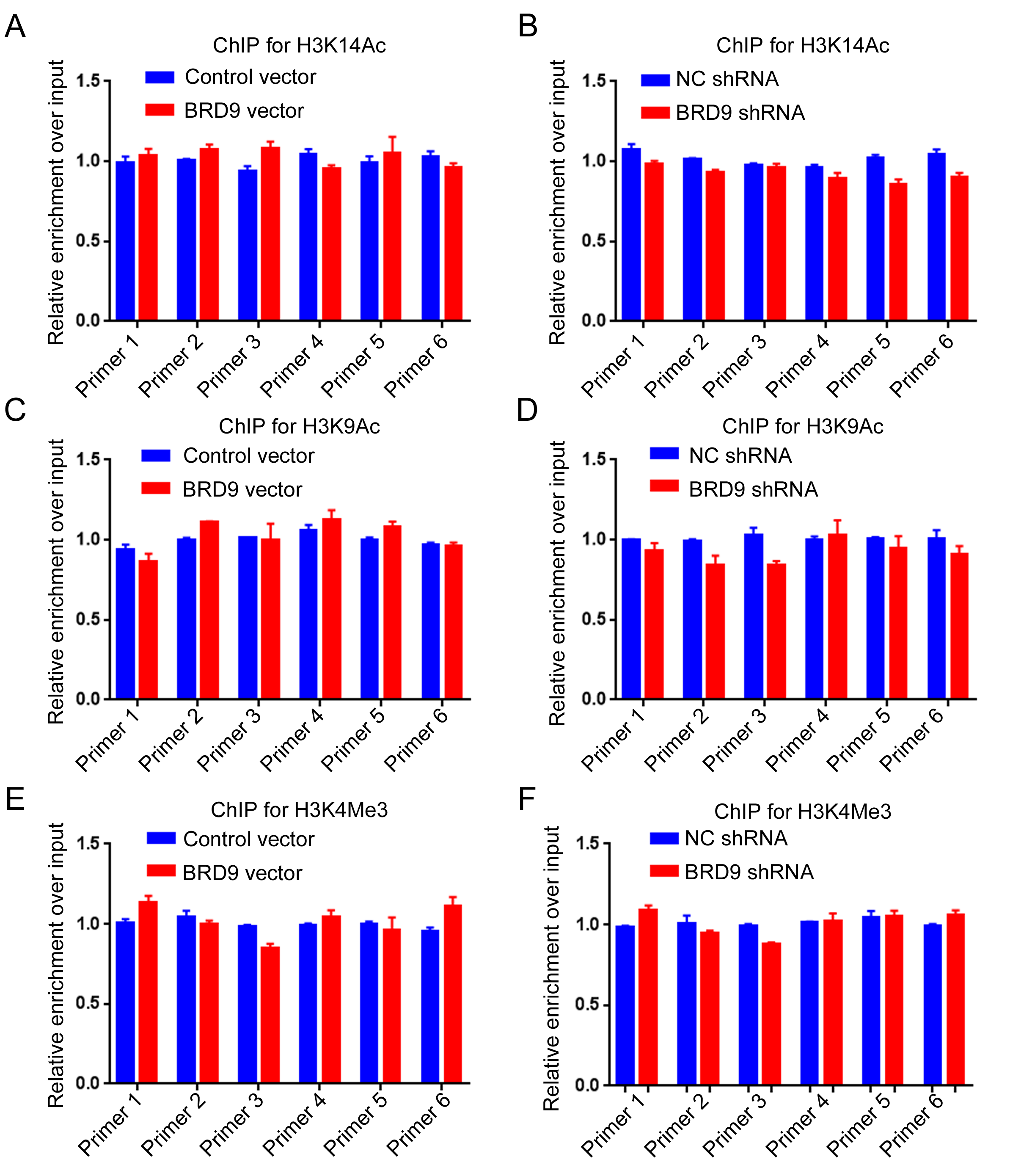

Supplement: Supplementary file 12 — Supplementary Figure 8 [file 41419_2020_2943_MOESM12_ESM.tif]

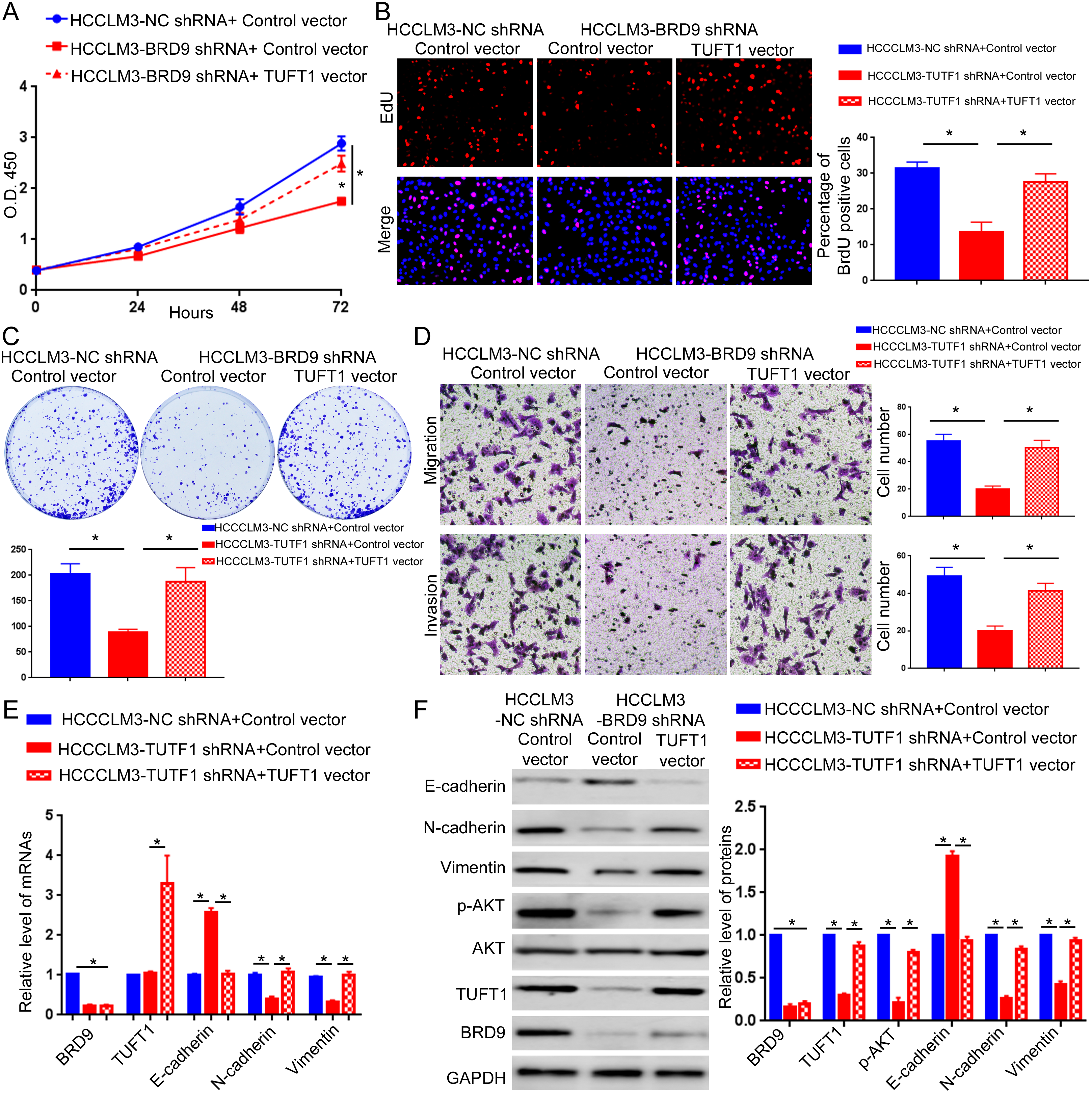

Supplement: Supplementary file 13 — Supplementary Figure 9 [file 41419_2020_2943_MOESM13_ESM.tif]
